# EM Adaptive LASSO—A Multilocus Modeling Strategy for Detecting SNPs Associated with Zero-inflated Count Phenotypes

**DOI:** 10.3389/fgene.2016.00032

**Published:** 2016-03-30

**Authors:** Himel Mallick, Hemant K. Tiwari

**Affiliations:** ^1^Department of Biostatistics, Harvard T. H. Chan School of Public Health, Harvard UniversityBoston, MA, USA; ^2^Program of Medical and Population Genetics, Broad Institute of MIT and HarvardCambridge, MA, USA; ^3^Section on Statistical Genetics, Department of Biostatistics, School of Public Health, University of Alabama at BirminghamBirmingham, AL, USA

**Keywords:** Zero-inflated Poisson, zero-inflated negative binomial, penalized regression, Poisson, negative binomial, genetic association studies, LASSO, adaptive LASSO

## Abstract

Count data are increasingly ubiquitous in genetic association studies, where it is possible to observe excess zero counts as compared to what is expected based on standard assumptions. For instance, in rheumatology, data are usually collected in multiple joints within a person or multiple sub-regions of a joint, and it is not uncommon that the phenotypes contain enormous number of zeroes due to the presence of excessive zero counts in majority of patients. Most existing statistical methods assume that the count phenotypes follow one of these four distributions with appropriate dispersion-handling mechanisms: Poisson, Zero-inflated Poisson (ZIP), Negative Binomial, and Zero-inflated Negative Binomial (ZINB). However, little is known about their implications in genetic association studies. Also, there is a relative paucity of literature on their usefulness with respect to model misspecification and variable selection. In this article, we have investigated the performance of several state-of-the-art approaches for handling zero-inflated count data along with a novel penalized regression approach with an adaptive LASSO penalty, by simulating data under a variety of disease models and linkage disequilibrium patterns. By taking into account data-adaptive weights in the estimation procedure, the proposed method provides greater flexibility in multi-SNP modeling of zero-inflated count phenotypes. A fast coordinate descent algorithm nested within an EM (expectation-maximization) algorithm is implemented for estimating the model parameters and conducting variable selection simultaneously. Results show that the proposed method has optimal performance in the presence of multicollinearity, as measured by both prediction accuracy and empirical power, which is especially apparent as the sample size increases. Moreover, the Type I error rates become more or less uncontrollable for the competing methods when a model is misspecified, a phenomenon routinely encountered in practice.

## Introduction

In genetic association studies, phenotypes are often measured in counts. For such count data, the standard methods for modeling the genotype–phenotype relationship include the Poisson and Negative Binomial regression models. The standard Poisson model assumes that the conditional variance of the phenotype is equal to the conditional mean. The Negative Binomial model generalizes the Poisson model by providing greater flexibility in explaining the relationship between the conditional variance and the conditional mean as compared to the Poisson model. However, in practice, it is not uncommon to observe excess zero counts as compared to what is expected based on either the Poisson or Negative Binomial model. As an example, in arthritis research, data are often collected in multiple joints within a person [i.e., number of hand joints with Radiographic Osteoarthritis (ROA)] or multiple sub-regions of a joint (i.e., number of sub-regions with cartilage loss in a knee; Zhang et al., 2010). As such, some of the phenotypes of interest in arthritis research are calculated based on a summary of individual integer-valued count measures (Teare et al., 2013), and therefore, it is highly plausible that they contain enormous number of zeroes due to the preponderance of zero counts in majority of patients. Some examples of such count phenotypes include the maximum number of pain sites (Holliday et al., 2010), total number of pain sites (Nicholl et al., 2011), and modified Larsen score (Teare et al., 2013), among others. In such situations, these two standard approaches [viz. the Poisson regression (PR) and the Negative Binomial (NB) regression] fail to take into account the added variability associated with the extraneous zero observations.

To account for the extra variability associated with the overabundant zero observations in count outcomes, various zero-inflated count models have been proposed in the literature, which include the Zero-inflated Poisson (ZIP) model (Lambert, 1992) and the Zero-inflated Negative Binomial (ZINB) model (Greene, 1994). These zero-inflated count models assume a latent mixture model consisting of two components: (i) a count component, which can be modeled as a Poisson or NB distribution and (ii) a degenerate zero component having a point mass at zero. Over the years, these two methods have become popular tools in statistical applications for analyzing count data. However, little is known about the utility of these approaches with respect to model misspecification. Most practitioners tend to use these tools without validating the data-generating mechanism. For instance, the most common statistical modeling technique used in analyzing zero-inflated count data is the ZIP regression. However, it has been established that the ZIP parameter estimates can be severely biased if the non-zero counts are over-dispersed in relation to the Poisson distribution (Greene, 1994). Therefore, blindly using a ZIP model in those situations can be misleading and inappropriate.

In addition to the above, there is also a relative paucity of literature on their implications in genetic association studies underlying zero-inflated count traits. In recent years, many Genome-wide association (GWA) studies have identified multiple genetic loci as being associated with rheumatoid arthritis (RA; Viatte et al., 2013a,b; Okada et al., 2014). However, the identified genetic effects tend to be moderate (OR ≤ 1.5) and explain only a small fraction (~16%) of the overall susceptibility (heritable and environmental; Viatte et al., 2013a,b). This is due to the inherent limitations of the single-marker analysis methods (simple univariate zero-inflated regression that analyzes one SNP at a time) commonly used in these GWA studies. These single-marker analyses may contribute weak but real effects on disease risks that are likely to be missed after taking the multiplicity adjustment into account (McCarthy et al., 2008). Moreover, single-marker analyses are doomed to low power for characterizing complex epistasis and gene-environment interactions (Hoggart et al., 2008; Li et al., 2011). Therefore, for unraveling the genetic architecture of complex diseases, it is necessary to accommodate multiple genetic variants and environmental covariates in a joint model, which is more powerful than traditional single-variant approaches. For such joint modeling, traditional statistical approaches such as the ZIP or ZINB might yield non-identifiable models (that is, having parameters that cannot be estimated) due to the presence of multiple highly linked variants. Moreover, with multiple genetic and environmental factors, there are many effects to estimate, most of which are likely to be zero or at least negligible, leading to high-dimensional sparse models, which lead to challenges in estimation, prediction, and interpretation.

Penalized regression methods provide a useful alternative for performing multi-SNP modeling. In penalized regression framework, a large number of possibly correlated variables and their interactions can be analyzed utilizing a single model (Szymczak et al., 2009). In statistical literature, variable selection has long been an active research area. Over the years, various penalized regression methods have been proposed for variable selection, which include the bridge regression (Frank and Friedman, 1993), Least Absolute Shrinkage And Selection Operator (LASSO; Tibshirani, 1996), Smoothly Clipped Absolute Deviation (SCAD; Fan and Li, 2001), Elastic Net (Zou and Hastie, 2005), Adaptive LASSO (Zou, 2006), and Minimax Concave Penalty (MCP; Zhang, 2010), among others. Generally, these methods are able to handle high-dimensional data in a computationally efficient manner by providing parsimonious model by simultaneous effect estimation and variable selection. However, most of these penalty functions are developed in the context of linear regression, survival models, and generalized linear models (GLMs), and it is not straightforward to adapt them to zero-inflated count phenotypes. This is due to the fact that variable selection is much more challenging for zero-inflated count models compared to linear, generalized linear, or survival models, as there are two separate model components contributing to the count outcomes.

Very recently, several authors have proposed penalized regression approaches for variable selection in zero-inflated count models. Among them, Buu et al. (2011) proposed an one-step SCAD estimator for the ZIP regression. Zeng et al. (2014) proposed an adaptive LASSO estimator for the ZINB regression models. However, both these procedures rely on Taylor approximation algorithm, which is known to be numerically instable if the Hessian matrix cannot be obtained accurately (Zeng et al., 2014). As an alternative, the well-known expectation-maximization (EM) algorithm provides a more natural and appropriate choice for estimating the parameters in zero-inflated count models. To this end, Tang et al. (2014) recently implemented an EM algorithm coupled with the adaptive LASSO estimator for the ZIP regression. Wang et al. (2014) developed EM algorithms for various penalized methods (viz. LASSO, MCP, and SCAD) for the ZIP regression. Wang et al. (2015) further extended their methods (viz. LASSO, MCP, and SCAD) to the ZINB regression. Some of these authors also established the so-called “oracle” property of their proposed estimators (i.e., they perform as well as if the “true” underlying model was given in advance). In this paper, along the same lines, we consider a more flexible penalized regression method for fitting the ZINB. More specifically, we propose an EM algorithm coupled with an adaptive LASSO estimator for the ZINB regression. While, Wang et al. (2015) considered a single shrinkage parameter for all the variables, we consider an improved version where different amount of shrinkage are allowed for different parameters. The idea is similar to Zou (2006), and with this formulation, the “oracle” property, consisting of both consistency in variable selection and estimation of non-zero coefficients, can be established, following arguments in either Tang et al. (2014) or Buu et al. (2011) (a proof is included in the Supplementary File). As noted by Zou (2006) in Remark 2 of his original adaptive LASSO paper: “As the sample size grows, the weights for zero-coefficient predictors get inflated (to infinity), whereas the weights for non-zero coefficient predictors converge to a finite constant. Thus we can simultaneously unbiasedly (asymptotically) estimate large coefficient and small threshold estimates.” As it will be clear from the simulation studies, the proposed method is more powerful than the existing methods with well-controlled Type I error, under a variety of disease models and linkage disequilibrium (LD) patterns. Moreover, the proposed method significantly outperforms the existing methods in terms of prediction accuracy.

The rest of the paper is organized as follows. In Section Methods, we introduce and discuss the proposed adaptive LASSO estimator for the ZINB regression. In Section Simulations, we describe our simulation experiments. In Section Results, we present the results of the simulation study comparing the proposed method with the existing methods. In Section Discussion, we provide guidelines to researchers and practitioners alike for using zero-inflated count models arising in genetic association studies including some concluding remarks as well as directions for future research.

## Methods

Suppose that a population-based association study consists of *n* unrelated individuals, phenotyped for a quantitative count trait and genotyped for a number of genetic variants in one or multiple candidate genes or genomic regions. We assume that there are *k* genetic variants. We denote the genotypes of variant *j* by *A*_*j*_*A*_*j*_, *A*_*j*_*a*_*j*_, or *a*_*j*_*a*_*j*_, where *a*_*j*_ is the minor allele with the observed frequency *p*_*j*_, *j* = 1, …, *k*. For an additive model, *x*_*ij*_ = 0, 1, or 2 for *A*_*j*_*A*_*j*_, *A*_*j*_*a*_*j*_, or *a*_*j*_*a*_*j*_, and for a dominant model, *x*_*ij*_ = 0 or 1 for *A*_*j*_*A*_*j*_ or *A*_*j*_*a*_*j*_ or *a*_*j*_*a*_*j*_, respectively. We also assume that *m* relevant non-genetic variables are measured for each individual, which will be included as covariates in the model. The observed values of the response variable are denoted by *y* = (*y*_1_, *y*_2_, …, *y*_*n*_)′. The GLM (McCullagh and Nelder, 1989) relates the linear predictor to the mean of the response variable via a link function as

(1) $$g(E(y_{i}|X_{i}))=\beta_{0}+\sum_{j\;=\;1}^{m}x_{ij}^{c}\beta_{j}^{c}+\sum_{j\;=\;1}^{k}x_{ij}^{g}\beta_{j}^{g}\;≜\;X_{i}\beta ,i=1,\ldots ,n,$$

where *g* is a link function, β_0_ is the intercept, $x_{ij}^{c}$ and $x_{ij}^{g}$ represent observed values of the covariates and genetic variables respectively, the coefficients $\beta_{j}^{c}$ and $\beta_{j}^{g}$ respectively denote non-genetic and genetic effects, *X*_*i*_ contains all variables and β is the vector of all coefficients and the intercept. For simplicity, we denote $X_{i}={\left(1,x_{i1},x_{i2},\ldots ,x_{ip}\right)}^{\prime }$, *i* = 1, …, *n* and $\beta ={\left(\beta_{0},\beta_{1},\ldots ,\beta_{p}\right)}^{\prime }$ where *p* = *m* + *k* is the total number of variables.

The likelihood function of a GLM can be expressed as

(2) $$p(y|X\beta ,\phi )=\prod_{i\;=\;1}^{n}p(y_{i}|X_{i}\beta ,\phi ),$$

where the distribution *p*(*y*_*i*_|*X*_*i*_β, ϕ) can take various forms, including Normal, Gamma, Binomial, Negative Binomial (for a known dispersion parameter), and Poisson distributions and ϕ is a dispersion parameter. The standard algorithm for fitting GLMs is the iterative weighted least squares (IWLS). Given the current estimates of the parameters $\left(\hat{\beta },\hat{\phi }\right)$, the IWLS algorithm constructs the pseudo-response *z*_*i*_ and the pseudo-weight *w*_*i*_ for each data point *y*_*i*_ as

(3) $$z_{i}={\hat{\eta }}_{i}-L^{\prime }(y_{i}|{\hat{\eta }}_{i})/L^{\prime \prime }(y_{i}|{\hat{\eta }}_{i}),w_{i}=-L^{\prime \prime }(y_{i}|{\hat{\eta }}_{i}),$$

and approximates the likelihood *p*(*y*_*i*_|*X*_*i*_β, ϕ) by the weighted normal likelihood as

(4) $$p(y_{i}|X_{i}\beta ,\phi )\approx N(z_{i}|X_{i}\beta ,w_{i}^{-1}\phi ),$$

where ${\hat{\eta }}_{i}=X_{i}\hat{\beta },L\left(y_{i}|{\hat{\eta }}_{i}\right)=\log p\left(y_{i}|X_{i}\hat{\beta },\hat{\phi }\right),L^{\prime }\left(y_{i}|{\hat{\eta }}_{i}\right)=dL\left(y_{i}|\eta_{i}\right)∕d\eta_{i}$ and $L^{\prime \prime }\left(y_{i}|\eta_{i}\right)=d^{2}L\left(y_{i}|\eta_{i}\right)∕d\eta_{{}_{i}}^{2}$. The parameters (β, ϕ) are then updated by solving the weighted normal linear regression Equation (4) using the weighted least squares.

The GLM includes the Poisson regression (PR), for which the corresponding probability mass function is given by

(5) $$f(y;\mu )=\frac{\mu^{y}e^{-\mu }}{y!},$$

where *E*(*y*) = *Var*(*y*) = μ and *g*(μ) = log(μ). An alternative to the PR is the NB regression, which is a flexible generalization of the PR, and is known to better handle the overdispersion present in a count data. The probability mass function for the two-parameter NB distribution is given by

(6) $$f(y;\theta ,\mu )=\frac{\Gamma (\theta +y)}{y!\Gamma (\theta )}{\left(\frac{\mu }{\mu +\theta }\right)}^{y}{\left(\frac{\theta }{\mu +\theta }\right)}^{\theta }\text{​},$$

Where $E\left(y\right)=\mu ,Var\left(y\right)=\mu +\frac{\mu^{2}}{\theta },$ and *g*(μ) = log(μ). Note that, we do not have a dispersion parameter for the PR; that is, ϕ is fixed at 1. On the other hand, the dispersion parameter for the NB regression is given by ϕ = θ. The parameter θ can be estimated from the data; however, the EM algorithm can be slow to converge. For simplicity, we assume θ = 1 in this article.

### Zero-inflated poisson (ZIP) model

Following Lambert (1992), a ZIP model for the count phenotype can be written as follows

(7) $$\begin{array}{ll} & y_{i}=0\text{ with probability }\pi_{i}\\ & y_{i}\sim \text{ Poisson(}\mu_{i}\text{) with probablity }(1-\pi_{i}) \end{array}\}\;i=1,\ldots ,n$$

The probability mass function of *y*_*i*_ can be written as follows

(8) $$\begin{array}{l} P(y_{i}=0)=\pi_{i}+(1-\pi_{i})e^{-\mu_{i}},\\ P(y_{i}=j)=(1-\pi_{i})\frac{e^{-\mu_{i}}\mu_{i}^{j}}{j!},j=1,\;2,\ldots \end{array}$$

It can be observed that Equation (8) reduces to the general PR when π_*i*_ = 0. Also, when π_*i*_ > 0, $P\left(y_{i}=0\right)>{\text{e}}^{-\mu_{i}}$, which indicates zero-inflation.

### Zero-inflated negative binomial (ZINB) model

Similarly, following Greene (1994), a ZINB model for the count phenotype can be written as follows

(9) $$\begin{array}{ll} & y_{i}=0\text{ with probablity }\pi_{i}\\ & y_{i}\sim \text{NB}(\mu_{i},\theta )\text{ with probablity }(\text{1}-\pi_{i}) \end{array}\}\;i=1,\ldots ,n$$

The probability mass function of *y*_*i*_ can be written as follows

(10) $$\begin{array}{l} P(y_{i}=0)=\pi_{i}+(1-\pi_{i}){\left(\frac{\theta }{\theta +\mu_{i}}\right)}^{\theta },\\ P(y_{i}=j)=(1-\pi_{i})\frac{\Gamma (\theta +y_{i})}{y_{i}!\Gamma (\theta )}{\left(\frac{\mu_{\text{i}}}{\theta +\mu_{\text{i}}}\right)}^{y}{\left(\frac{\theta }{\theta +\mu_{i}}\right)}^{\theta },\\ \;j=1,\;2\ldots \end{array}$$

Again, it can be observed that Equation (10) reduces to the general NB regression when π_*i*_ = 0. Also, when π_*i*_ > 0, $p\left(y_{i}=0\right)>{\left(\frac{\theta }{\theta \;+\;\mu_{i}}\right)}^{\theta }$, which indicates zero-inflation. For both these models, the parameters π_*i*_ and μ_*i*_ can be modeled (Lambert, 1992; Greene, 1994) by a logistic regression model (for the zero component) and a log-linear model (for the count component) respectively as follows

(11) $$\begin{array}{ll} & \text{log}\left(\frac{\pi_{i}}{1-\pi_{\text{i}}}\right)={\text{X}}_{i}\gamma ,\\ & \log(\mu_{i})={\text{X}}_{\text{i}}\beta , \end{array}\}$$

where *X*_*i*_ contains all variables corresponding to the *i*th observation, *i* = 1, …, *n* and β and γ are the vectors of all genetic and non-genetic coefficients and the intercept corresponding to the count and zero components respectively. In practice, we may have a different set of predictors corresponding to each of these two components, for which Equation (11) can be adapted accordingly. The ZINB model is more appropriate to incorporate extra over dispersion not accounted for through zero-inflation by the ZIP model (Greene, 1994). Therefore, in this article, we primarily focus on the ZINB regression and investigate the effect of model misspecification when the underlying data-generating mechanism is indeed ZINB. For variable selection, we propose the EM adaptive LASSO method for the ZINB regression, which we describe next.

### The adaptive LASSO model

One major challenge in analyzing genetic data with zero-inflated count phenotypes is the problem of variable selection, as most of the existing methods such as the subset selection or step-wise procedures are computationally tedious for analyzing such datasets, especially when the number of variables is large and/or there exists collinearity. In addition, variable selection for mixture models is much more complicated than that for non-mixture models. To meet these challenges, we propose the EM Adaptive LASSO (AL) estimator by modifying the EM algorithm developed by Wang et al. (2015) for their LASSO estimator, which is implemented in the ***R*** (R Core Team, 2015) *package* ***mpath*** (Wang, 2015). With this approach, we are able to conduct simultaneous model selection and stable effect estimation in the presence of multicollinearity, which reduces the burden of multiple testing. The log-likelihood function for the ZINB regression model (assuming θ = 1) is given by

(12) $$\begin{array}{l} L(\beta ,\gamma )=\sum_{y_{i}\;=\;0}\log\left[\pi_{i}+(1-\pi )\left(\frac{1}{1+\mu_{i}}\right)\right]\\ \;+\sum_{y_{i}>0}\log\left[(1-\pi_{i}){\left(\frac{\mu_{i}}{1+\mu_{i}}\right)}^{y}\left(\frac{1}{1+\mu_{i}}\right)\right]\text{​}, \end{array}$$

where μ_*i*_ = exp(*X*_*i*_β), and $\pi_{i}=\frac{\exp\left(X_{i}\gamma \right)}{1+\exp\left(X_{i}\gamma \right)},i=1,\ldots ,n.$

For variable selection, we consider a penalized ZINB model with the adaptive LASSO penalty, which results from the following regularization problem

(13) $$Q(\beta ,\gamma )=-2\log L(\beta ,\gamma )+\lambda_{1}\sum_{j\;=\;1}^{p}w_{1j}\left|\beta_{j}\right|+\lambda_{2}\sum_{j\;=\;1}^{p}w_{2j}\left|\gamma_{j}\right|,$$

where $w_{1}={\left(w_{11},\ldots ,w_{1p}\right)}^{\prime }$ and $w_{2}={\left(w_{21},\ldots ,w_{2p}\right)}^{\prime }$ are known weight vectors, which are usually taken as the reciprocal of the unpenalized estimates obtained by maximizing Equation (12), i.e., $w_{1}=\frac{1}{\left|{\hat{\beta }}_{\text{MLE}}\right|}$ and $w_{2}=\frac{1}{\left|{\hat{\gamma }}_{\text{MLE}}\right|}$. Note that, when *w*_1**j**_ = 1, *w*_2**j**_ = 1, *j* = 1, …, *p*, it reduces to the LASSO estimator (Wang et al., 2015).

### The EM algorithm

We formulate Equation (13) as a missing data problem and solve the problem by EM algorithm. To this end, we assume that there are two distinct processes driving the zeros in the count phenotype of interest: one is the result of a zero process, and the other one is a part of a counting process (NB). We consider the following latent variables: *z*_*i*_ = 1 if *y*_*i*_ is from the zero state and *z*_*i*_ = 0 if *y*_*i*_ is from the count (NB) state, *i* = 1, …, *n*. Since, *z*_*i*_'s are not observable, we treat them as the “missing data”. With these missing variables, the complete-data log-likelihood function is given by

(14) $$\begin{array}{l} PL(\beta \text{,}\gamma )=\sum_{i\;=\;1}^{n}[z_{i}X_{i}\gamma -\log(1+{\text{exp(X}}_{i}\gamma ))\\ \;+\;(1-z_{i})\{y_{i}X_{i}\beta -(y_{i}+1)\log\left(1+X_{i}\beta \right)\}] \end{array}$$

The penalized ZINB model with the complete data log-likelihood is given by

(15) $$Q^{*}(\beta ,\gamma )=-2\log PL(\beta ,\gamma )+\lambda_{1}\sum_{j\;=\;1}^{p}w_{1j}\left|\beta_{j}\right|+\lambda_{2}\sum_{j\;=\;1}^{p}w_{2j}\left|\gamma_{j}\right|,$$

where at the E step of the algorithm, we compute the expectation of the complete-data log-likelihood by replacing *z*_*i*_'s by their conditional expectation given the observed data and current estimates, and in the M step, we estimate the coefficients by minimizing the expected penalized complete-data log-likelihood Equation (15). Starting with an initial guess of the coefficients, the algorithm proceeds as follows:

1) E Step
  $${\hat{z}}_{i}^{\left(t\right)}=\{\begin{array}{cc} {\left(1+\left[\frac{{\text{exp(-X}}_{i}{\hat{\gamma }}^{\left(t\right)}}{1+{\text{exp(X}}_{i}{\hat{\beta }}^{\left(t\right)}}\right]\right)}^{-1} & \text{if }{\text{y}}_{i}=0\\ 0 & \text{if }{\text{y}}_{i}>0 \end{array}$$
2) M Step: Minimize the following objective function
  (16) $$\begin{array}{l} Q^{*}(\beta ,\gamma |\beta^{\left(t\right)},\gamma^{\left(t\right)})=-2E(PL(\beta ,\gamma |y,z)|\beta^{\left(t\right)},\gamma^{\left(t\right)}\\ \;+\;\lambda_{1}\sum_{j\;=\;1}^{p}w_{1j}\left|\beta_{j}\right|+\lambda_{2}\sum_{j\;=\;1}^{p}w_{2j}\left|\beta_{j}\right| \end{array}$$
3) Repeat 1 and 2 until convergence, *t* = 1, 2….
  Note that, Equation (16) can be written as a sum of two components as follows
  $$Q^{*}(\beta ,\gamma |\beta^{\left(t\right)},\gamma^{\left(t\right)})=Q_{1}{}^{*}(\beta |\beta^{\left(t\right)},\gamma^{\left(t\right)})+Q_{2}{}^{*}(\gamma |\beta^{\left(t\right)},\gamma^{\left(t\right)}),$$
  where $\begin{array}{l} Q_{1}^{*}(\beta |\beta^{\left(t\right)},\gamma^{\left(t\right)})=-2[\sum_{i\;=\;1}^{n}\left(1-{\hat{z}}_{i}^{\left(t\right)}\right)\{y_{i}X_{i}\beta -(y_{i}\\ +1)\log(1+X_{i}\beta )\;\}]+\lambda_{1}\sum_{j\;=\;1}^{p}w_{1j}\left|\beta_{j}\right|, \end{array}$ and $\begin{array}{l} Q_{2}^{*}(\gamma |\beta^{\left(t\right)},\gamma^{\left(t\right)})=-2\left[\sum_{i\;=\;1}^{n}{\hat{z}}_{i}^{\left(t\right)}X_{i}\gamma -\log(\left(1+\text{exp(}X_{i}\gamma )\right)\right]\\ +\;\lambda_{2}\sum_{j\;=\;1}^{p}w_{2j}\left|\gamma_{j}\right|. \end{array}$.

The first component $Q_{1}^{*}\left(\beta |\beta^{\left(t\right)},\gamma^{\left(t\right)}\right)$ is a weighted penalized NB log-likelihood and the second component $Q_{2}^{*}(\gamma |(\beta^{\left(t\right)},\gamma^{\left(t\right)})$ is a penalized logistic log-likelihood, both of which belong to the GLM family (without the penalties). Due to the form of the objective function, these two components can be minimized separately, using computationally efficient coordinate descent algorithms developed for GLM (Friedman et al., 2010). The details of this algorithm, which utilizes the IWLS algorithm described above (Friedman et al., 2010), can be found in Friedman et al. (2010). Recently, Wang et al. (2015) adapted this procedure for their LASSO estimator in the ***R*** package ***mpath***. Here we further improve the algorithm by allowing different penalties for each of the variables. The variable-specific parameters provide a way to pool the information among the variables. Naturally, for the unimportant variables, we should put larger penalty parameters on their corresponding coefficients. Although the general LASSO estimator may not be consistent (Zou, 2006), with appropriately chosen data-dependent weights *w*_1*j*_ 's and *w*_2*j*_ 's, we are able to warrant the “oracle property” for the adaptive LASSO estimator. Therefore, the adaptive LASSO estimates are much more attractive than the general LASSO estimates. In high-dimensional scenarios, these weights may not available, due to the presence of data collinearity. In those situations, we make use of the reciprocal of the ridge-penalized estimates (Hoerl and Kennard, 1970) as weights, which are usually available.

### Selection of tuning parameters

For selecting the tuning parameters, following Wang et al. (2015), we first construct a solution path based on the paired shrinkage parameters. Then, we choose the final estimates as determined by the minimum BIC information defined as

BIC=−2logL+dlog(n),

where *d* is the number of non-zero parameters in the model and *n* is the sample size. It should be noted that the BIC is asymptotically consistent in terms of model selection (Burnham and Anderson, 2004; Dziak et al., 2005; Naik et al., 2007; Wang et al., 2007, 2009), which means that the probability of the selected model being the true model converges to 1 as the sample size increases.

### Practical implementation

To implement our algorithm, we make use of the ***R*** packages ***pscl*** (Zeileis et al., 2008) and ***mpath***. First, we obtain the maximum likelihood estimates by running the ***glm.nb*** function in the ***R*** package ***pscl*** for fitting ZINB regression models, and then we supply the weights (reciprocal of the maximum likelihood estimates) to the ***zipath*** function in the ***R*** package ***mpath*** through the argument ***penalty.factor***. In situations when the maximum likelihood estimates are not available, we make use of the reciprocal of the ridge-penalized estimates as weights, available from the ***R*** package ***mpath***.

## Simulations

In this section, we conduct extensive simulation experiments to evaluate the finite sample performance of the proposed method, and compare the results with several existing methods with respect to model misspecification and variable selection.

### Simulations with simulated genotypes

We generate the simulated data as in Wang and Elston (2007). The genotype data (containing *k* SNPs) is generated as follows:

1) First, we generate a latent vector $Z=(Z_{1},Z_{2},\ldots ,Z_{k}{)}^{\prime }$ from a multivariate normal (MVN) distribution with mean 0 and AR (1) covariance matrix (power decay correlation), i.e., $Cov\left(Z_{i},Z_{j}\right)=\rho^{\left|i-j\right|},i\neq j.$
2) Second, we dichotomize *Z* into a haplotype, say $H_{1}={\left(h_{11},\ldots ,h_{1k}\right)}^{\prime }$ with *h*_1**j**_ = *I*(*Z*_*j*_ < *MAF*), where the MAF is fixed at 5%.
3) Similarly, we generate another independent haplotype *H*_2_. Combining the two haplotypes (H_1_ + H_2_) we obtain an individual's genotype.

Based on the genotype data, we construct our design matrix *X* assuming an additive model and generate the phenotype of interest according to the model in Equation (11), where the phenotype follows a ZINB distribution. For the null case, we assign β = 0; for the non-null case with varying association strengths, the effect sizes (for the count model) are determined so that all causal SNPs contribute the same marginal variance *h*_*j*_^2^ to the linear predictor, where *h*_*j*_^2^ is defined as *h*_*j*_^2^ = 2 ^*^ *MAF* ^*^ (1–*MAF*) ^*^ β_*j*_^2^. We assume that there are *k* = 50 SNPs among which 5 are assumed to be causal (in particular, we assume the 1st, 11th, 21st, 31st, and 41st SNP to be causal). To ensure that the overall effect of all variants is reasonably low, we fix the sum of their marginal variances *h* = ∑ *h*_*j*_^2^ at 0.05, 0.10, 0.15, 0.20, 0.25, and 0.30 under the alternative hypothesis, assuming equal marginal variance for each causal SNP. The simulation scenarios are also presented in Table 1. For simplicity, we consider γ = 0 i.e., an intercept-only model is assumed for the zero component. The zero inflation is assumed to be 50% across all simulation examples. We consider three linkage disequilibrium patterns: (i) Independent SNPs (ρ = 0), (ii) SNPs in moderate LD (ρ = 0.5), and (iii) SNPs in high LD (ρ = 0.9). For each situation, 1000 replicated datasets are simulated. For each of these simulation scenarios, we consider three sample sizes: small (*n* = 200), moderate (*n* = 500), and large (*n* = 1000).

**Table 1:** **Simulation Designs for Varying Marginal Variances**.

**No. of SNPs**	**No. of Causal SNPs**	**Marginal Variance**	**Sum of Marginal Variances**	**MAF %**
50	5	0.01	0.05	5
50	5	0.02	0.10	5
50	5	0.03	0.15	5
50	5	0.04	0.20	5
50	5	0.05	0.25	5
50	5	0.06	0.30	5

### Simulations with real genotypes

As one anonymous reviewer suggested, to mimic real data and to be as practical as possible, the best way to validate and study the properties of the proposed method will be to take real sequence data obtained from many individuals and simulate phenotypes based on variants in those sequences, making assumptions only about genetic effects of variants. To this end, we used the genotypes (i.e., *X*_*i*_'s) from a real data to generate a zero-inflated NB outcome, and performed additional simulation studies by taking advantage of the real genotypes of variants in a large real dataset.

Kaklamani et al. (2008) investigated the association of genetic variants of the adiponectin (ADIPOQ) and adiponectin receptor 1 (ADIPOR1) genes with colorectal cancer risk in a large case-control study. This case-control study included a total of 441 patients with a diagnosis of colorectal cancer and 658 unrelated controls. All cases and controls were white and of Ashkenazi Jewish ancestry and from New York. Information regarding gender, current age for controls, and age at colorectal cancer diagnosis for cases was recorded. Five haplotype-tagging SNPs were selected to capture variations in the major blocks in each of genes ADIPOQ and ADIPOR1. The selected SNPs have MAF above 10% and show low proportions of missing genotypes (from 0.3 to 3%). The genotypic frequencies of the 10 SNPs are reported in Table 1 of Yi et al. (2011). For the missing genotypes, following previous analyses (Yi et al., 2011), we filled in the variables using the expectation of the observed values in that marker. As before, the zero inflation is assumed to be 50% across all examples, with an intercept-only model for the zero component. We assume that there are 5 causal effects among the 20 main effects under codominant model. In particular, we assume the 1st, 5th, 9th, 13th, and 17th genetic effect to be causal. As before, the sum of marginal variances (*h* = ∑ *h*_*j*_^2^) is fixed at 5, 10, 15, 20, 25, and 30% under the alternative hypothesis, assuming equal marginal variance for each causal SNP. For each situation, 1000 replicated datasets are simulated.

Each generated data is analyzed using six methods, viz. the Poisson regression (PR), ZIP regression, Negative Binomial (NB) regression, ZINB regression, the LASSO method of Wang et al. (2015), and the proposed method (AL). To summarize the simulation results, we calculate the percentage of times a regression coefficient, and hence the corresponding variant, is found to be significant. Thus, this percentage is essentially power or type I error for that variant depending on whether the variant is truly associated or not associated. Therefore, the type I error in this context is the event of declaring a variant to be significant when it is not truly associated. For the unpenalized methods, we reject the null hypothesis that the genetic effect of an individual SNP equals to zero at the significance level of 5% with the False Discovery Rate (FDR) adjustment (Benjamini and Yekutieli, 2001). For the proposed adaptive LASSO (AL) procedure, the non-zero coefficients are known to have asymptotic normal distributions, which is due to the established oracle property of the estimator. Therefore, to test the non-zero estimated coefficients, we reject the null hypothesis that the genetic effect of an individual SNP equals to zero at the same significance level as before. It is to be noted that, we are not clear whether we can use the same significance testing framework for the non-zero coefficients in the LASSO procedure, as it is unknown whether the estimated non-zero LASSO coefficients follow an asymptotic normal distribution for the ZINB regression. Nevertheless, for a complete evaluation, we also compare the sensitivity and specificity of the proposed method with that of the LASSO method, where sensitivity and specificity refer to the proportion of true non-zero coefficients and true zero coefficients that are detected by each method.

## Results

### Power and type I error (simulated genotypes)

**Figure 1:**
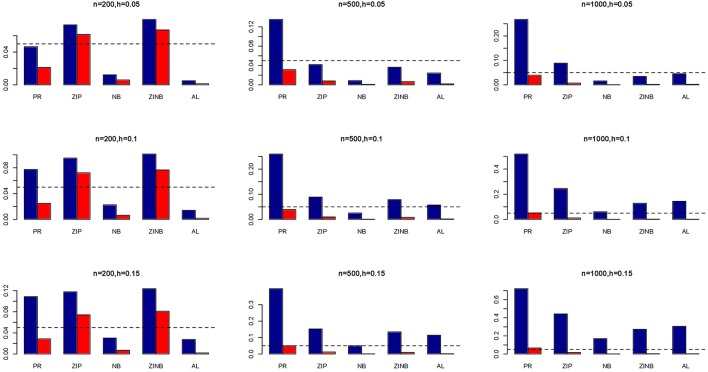
**Average Type I Error Rates (Red Bar) and Average Power (Blue Bar) from 1000 Replications for Independents SNPs (ρ = 0)**. Five methods are displayed from left to right: Poisson Regression (PR), Zero-inflated Poisson (ZIP) regression, Negative Binomial (NB) regression, Zero-inflated Negative Binomial (ZINB) regression, and adaptive LASSO (AL) penalized NB regression. Three sample sizes (*n* = 200, 500, 1000) (in rows) and three marginal variances (*h* = 0.05, 0.10, 0.15) (in columns) are presented.

**Figure 2:**
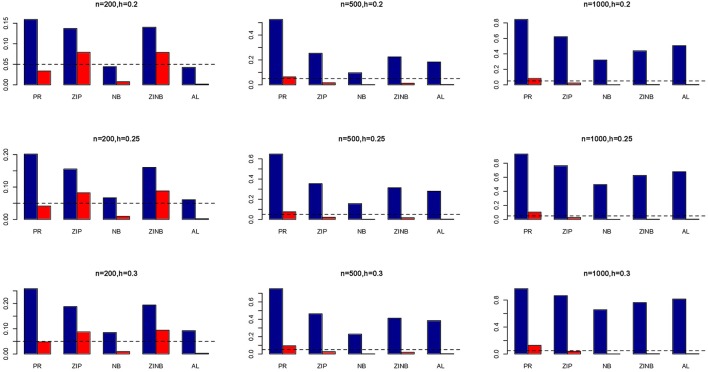
**Average Type I Error Rates (Red Bar) and Average Power (Blue Bar) from 1000 Replications for Independents SNPs (ρ = 0)**. Five methods are displayed from left to right: Poisson Regression (PR), Zero-inflated Poisson (ZIP) regression, Negative Binomial (NB) regression, Zero-inflated Negative Binomial (ZINB) regression, and adaptive LASSO (AL) penalized NB regression. Three sample sizes (*n* = 200, 500, 1000) (in rows) and three marginal variances (*h* = 0.20, 0.25, 0.30) (in columns) are presented.

**Figure 3:**
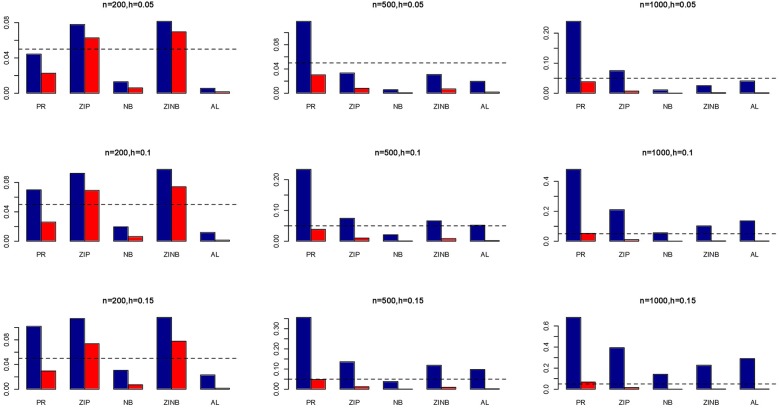
**Average Type I Error Rates (Red Bar) and Average Power (Blue Bar) from 1000 Replications for SNPs in Moderate LD (ρ = 0.5)**. Five methods are displayed from left to right: Poisson Regression (PR), Zero-inflated Poisson (ZIP) regression, Negative Binomial (NB) regression, Zero-inflated Negative Binomial (ZINB) regression, and adaptive LASSO (AL) penalized NB regression. Three sample sizes (*n* = 200, 500, 1000) (in rows) and three marginal variances (*h* = 0.05, 0.10, 0.15) (in columns) are presented.

**Figure 4:**
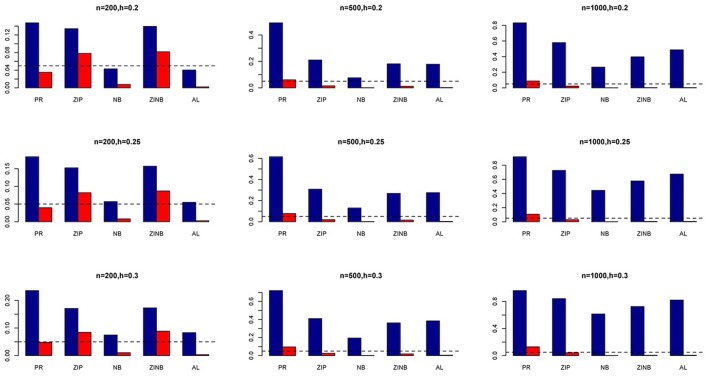
**Average Type I Error Rates (Red Bar) and Average Power (Blue Bar) from 1000 Replications for SNPs in Moderate LD (ρ = 0.5)**. Five methods are displayed from left to right: Poisson Regression (PR), Zero-inflated Poisson (ZIP) regression, Negative Binomial (NB) regression, Zero-inflated Negative Binomial (ZINB) regression, and adaptive LASSO (AL) penalized NB regression. Three sample sizes (*n* = 200, 500, 1000) (in rows) and three marginal variances (*h* = 0.20, 0.25, 0.30) (in columns) are presented.

**Figure 5:**
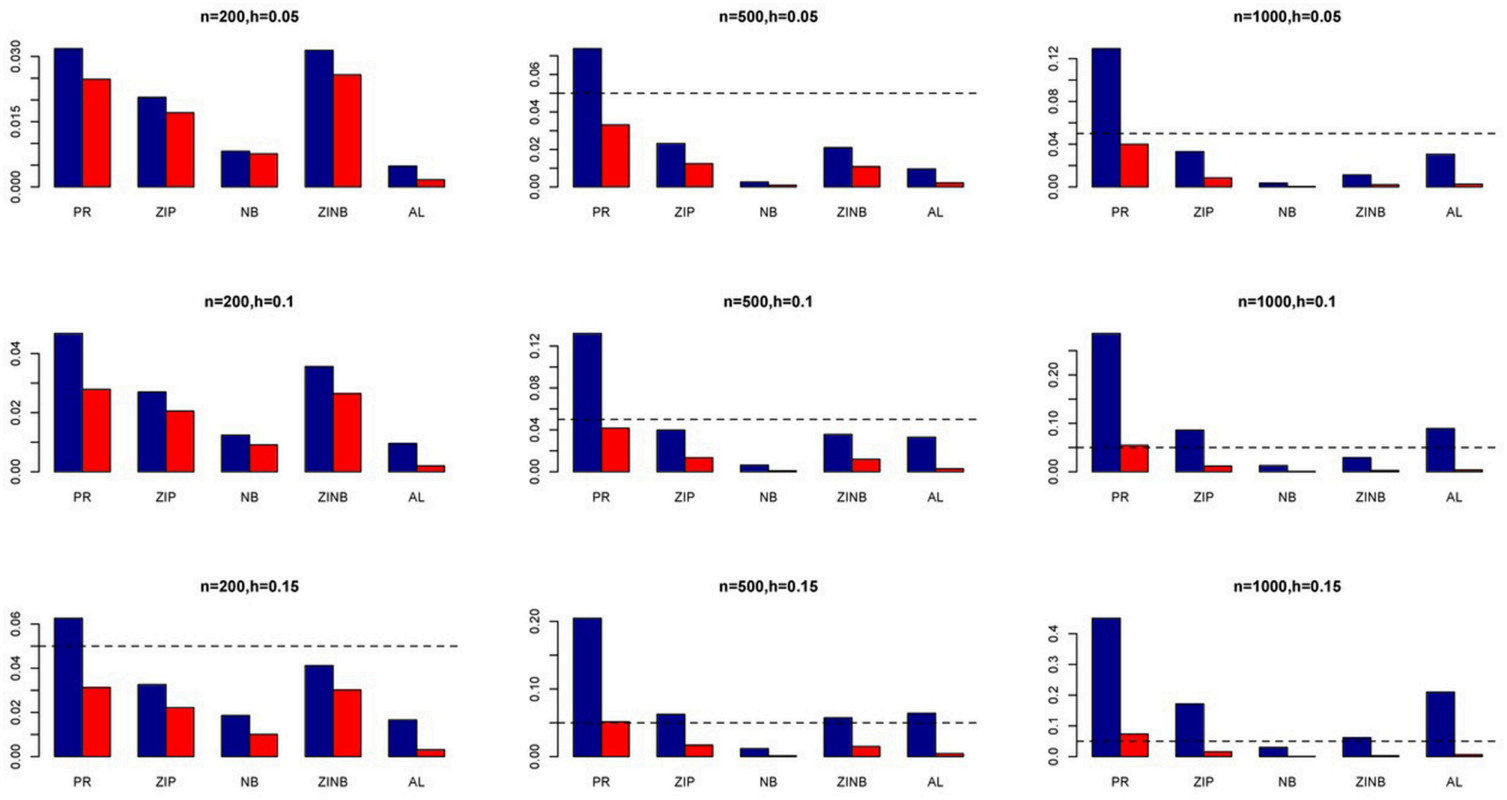
**Average Type I Error Rates (Red Bar) and Average Power (Blue Bar) from 1000 Replications for SNPs in High LD (ρ = 0.9)**. Five methods are displayed from left to right: Poisson Regression (PR), Zero-inflated Poisson (ZIP) regression, Negative Binomial (NB) regression, Zero-inflated Negative Binomial (ZINB) regression, and adaptive LASSO (AL) penalized NB regression. Three sample sizes (*n* = 200, 500, 1000) (in rows) and three marginal variances (*h* = 0.05, 0.10, 0.15) (in columns) are presented.

**Figure 6:**
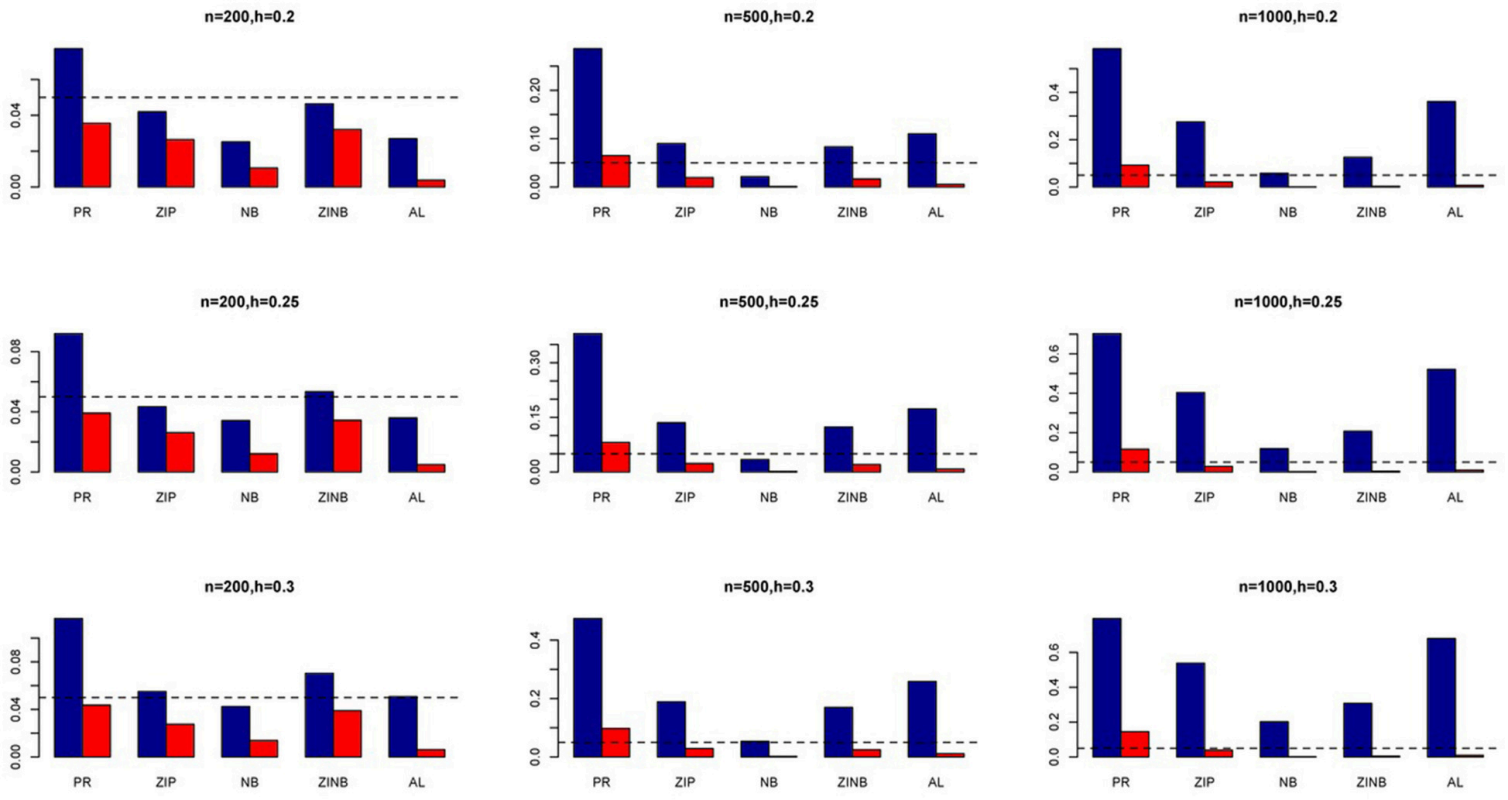
**Average Type I Error Rates (Red Bar) and Average Power (Blue Bar) from 1000 Replications for SNPs in High LD (ρ = 0.9)**. Five methods are displayed from left to right: Poisson Regression (PR), Zero-inflated Poisson (ZIP) regression, Negative Binomial (NB) regression, Zero-inflated Negative Binomial (ZINB) regression, and adaptive LASSO (AL) penalized NB regression. Three sample sizes (*n* = 200, 500, 1000) (in rows) and three marginal variances (*h* = 0.20, 0.25, 0.30) (in columns) are presented.

**Figure 7:**
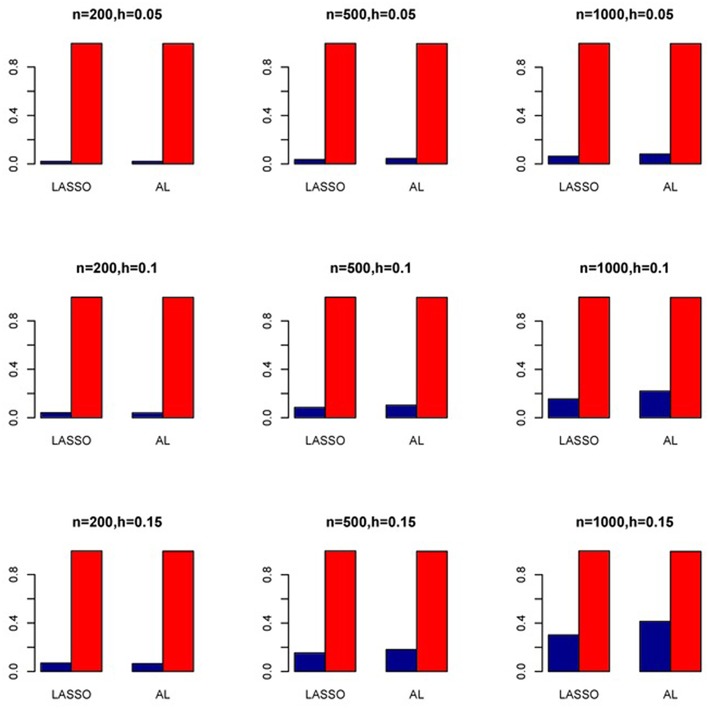
**Sensitivity (Red Bar) and Specificity (Blue Bar) from 1000 Replications for Independents SNPs (ρ = 0)**. Two methods are displayed: LASSO penalized NB regression (LASSO) and adaptive LASSO (AL) penalized NB regression. Three sample sizes (*n* = 200, 500, 1000) (in rows) and three marginal variances (*h* = 0.05, 0.10, 0.15) (in columns) are presented.

**Figure 8:**
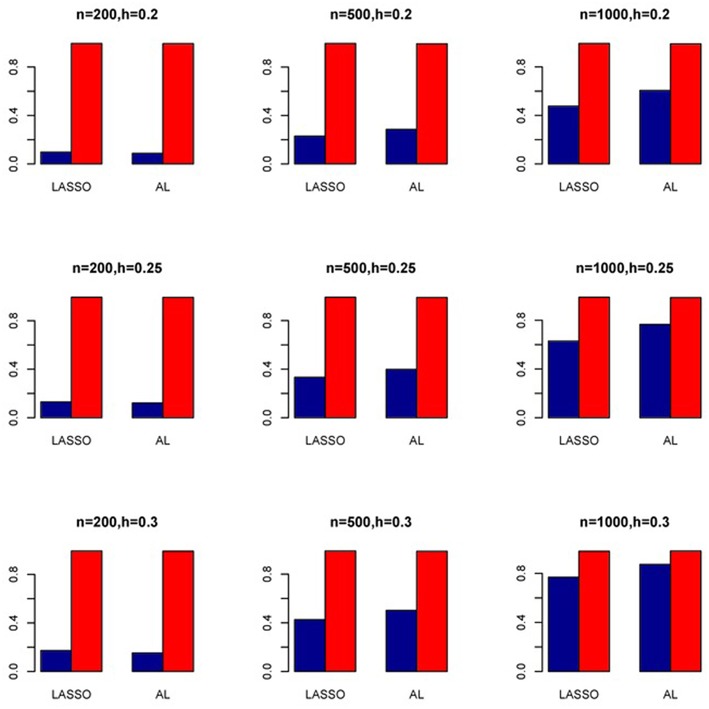
**Sensitivity (Red Bar) and Specificity (Blue Bar) from 1000 Replications for Independents SNPs (ρ = 0)**. Two methods are displayed: LASSO penalized NB regression (LASSO) and adaptive LASSO (AL) penalized NB regression. Three sample sizes (*n* = 200, 500, 1000) (in rows) and three marginal variances (*h* = 0.20, 0.25, 0.30) (in columns) are presented.

**Figure 9:**
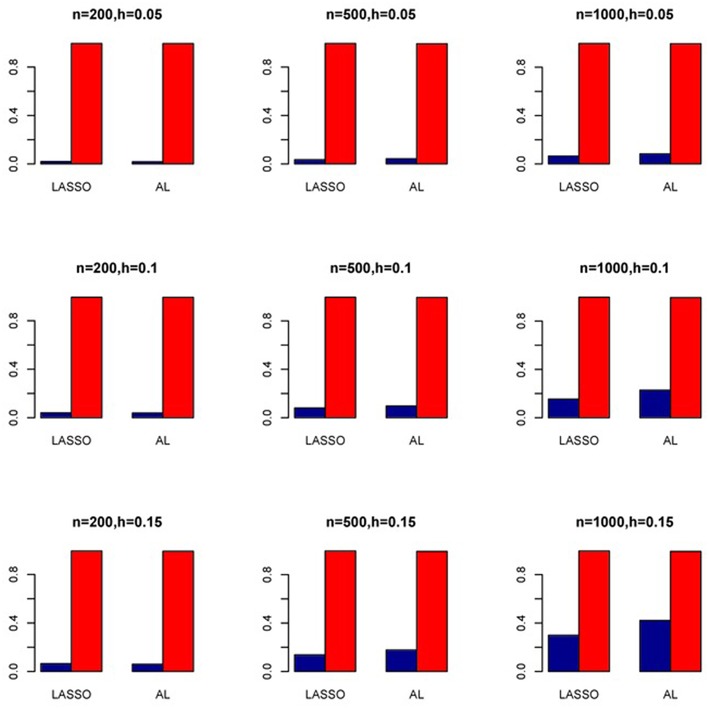
**Sensitivity (Red Bar) and Specificity (Blue Bar) from 1000 Replications for SNPs in Moderate LD (ρ = 0.5)**. Two methods are displayed: LASSO penalized NB regression (LASSO) and adaptive LASSO (AL) penalized NB regression. Three sample sizes (*n* = 200, 500, 1000) (in rows) and three marginal variances (*h* = 0.05, 0.10, 0.15) (in columns) are presented.

**Figure 10:**
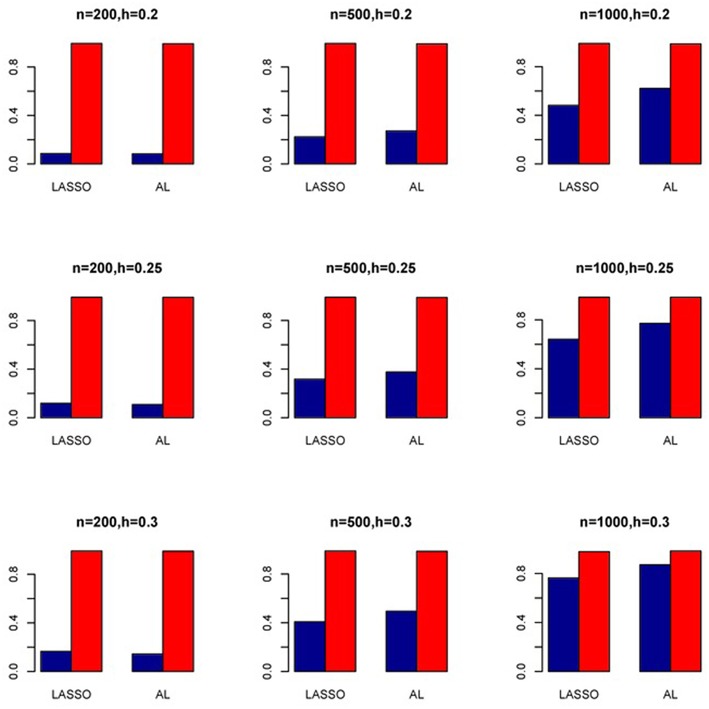
**Sensitivity (Red Bar) and Specificity (Blue Bar) from 1000 Replications for SNPs in Moderate LD (ρ = 0.5)**. Two methods are displayed: LASSO penalized NB regression (LASSO) and adaptive LASSO (AL) penalized NB regression. Three sample sizes (*n* = 200, 500, 1000) (in rows) and three marginal variances (*h* = 0.20, 0.25, 0.30) (in columns) are presented.

**Figure 11:**
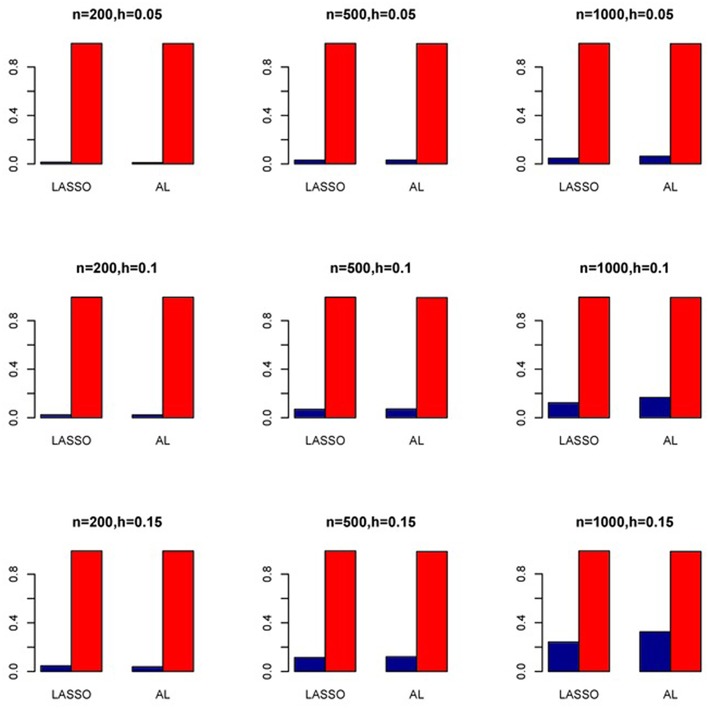
**Sensitivity (Red Bar) and Specificity (Blue Bar) from 1000 Replications for SNPs in High LD (ρ = 0.9)**. Two methods are displayed: LASSO penalized NB regression (LASSO) and adaptive LASSO (AL) penalized NB regression. Three sample sizes (*n* = 200, 500, 1000) (in rows) and three marginal variances (*h* = 0.05, 0.10, 0.15) (in columns) are presented.

**Figure 12:**
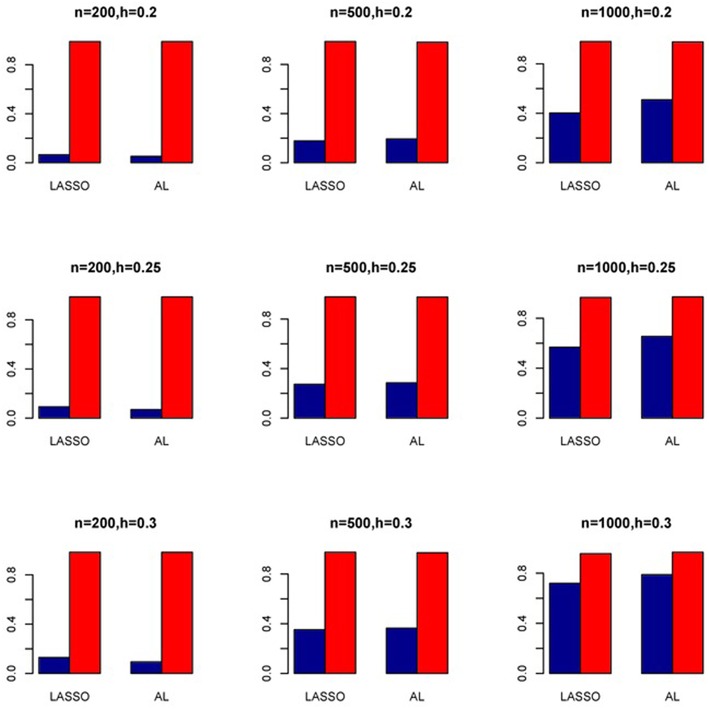
**Sensitivity (Red Bar) and Specificity (Blue Bar) from 1000 Replications for SNPs in High LD (ρ = 0.9)**. Two methods are displayed: LASSO penalized NB regression (LASSO) and adaptive LASSO (AL) penalized NB regression. Three sample sizes (*n* = 200, 500, 1000) (in rows) and three marginal variances (*h* = 0.20, 0.25, 0.30) (in columns) are presented.

The simulation results are summarized in Figures 1–6. Several observations are in order. First of all, for small sample sizes, the type I error rates for the PR and the ZIP models are high irrespective of the LD spectra of the variants. For moderate to large sample sizes, the type I error rate stays under control for the ZIP regression, although it remains uncontrolled for the PR. Second, the performance of the ZINB regression is surprisingly not better than the NB regression when the sample size is small. In fact, when *n* = 200, the type I error rate is out of control for the ZINB for all values of ρ, although not necessarily so for other scenarios. For moderate to large sample sizes, the ZINB performs better than the NB regression, as expected. Third, the proposed method is the most conservative among the methods considered, as it consistently maintains better control of Type I error rate as compared to other methods across all scenarios. Also, among the methods having well controlled type I error rates, the proposed AL procedure generally has higher power as compared to their non-penalized counterparts. Fourth, for high LD scenarios (ρ = 0.9), there is a systematic trend in the empirical powers for the methods considered. In particular, it follows the order: AL > ZINB > NB. Overall, the zero-inflated count models perform better than their regular counterparts in most of the scenarios. Fifth, we also evaluated sensitivity and specificity of the penalized regression methods for all the simulation scenarios described above (Figures 7–12). In all the situations, the proposed method consistently has better sensitivity as compared to the LASSO estimator, although both have similar specificity. As expected, the sensitivity drastically increases with larger sample sizes. Lastly, in our experience, the non-penalized methods perform slightly better than the penalized methods in terms of CPU time. However, the problem of non-identifiability of the parameter estimates frequently occurs (especially for small sample size) for non-penalized methods, whereas no such problem arises while using the penalized methods. In Table 2, we summarize the percentage of non-convergence of the methods when *n* = 200. It can be seen that the non-convergence issues only arise for ZIP, NB, and ZINB methods for small sample sizes. Therefore, we slightly overestimate the powers and type I error rates for these methods, as we compute these quantities only based on those replicates that converge.

**Table 2:** **Proportion of Non-identifiability of Parameter Estimates for Varying LD Structure for *n* = 200**.

**ρ**	**Marginal Variance**	**PR**	**ZIP**	**NB**	**ZINB**	**LASSO**	**AL**
ρ = 0	0.01	0	1.2	1	2.7	0	0
	0.02	0	0.6	0.7	1.6	0	0
	0.03	0	0.5	1.1	1.8	0	0
	0.04	0	0.7	1.7	1.7	0	0
	0.05	0	1	1.8	1.5	0	0
	0.06	0	1	2.3	2.2	0	0
ρ = 0.5	0.01	0	0.6	1.8	3.4	0	0
	0.02	0	0.9	1.9	1.9	0	0
	0.03	0	0.6	2.7	1.9	0	0
	0.04	0	0.9	1.2	2.8	0	0
	0.05	0	1.2	1.8	1.8	0	0
	0.06	0	1.5	2.6	2.5	0	0
ρ = 0.9	0.01	0	8.9	2.2	7.8	0	0
	0.02	0	10	2.5	8.2	0	0
	0.03	0	9	3.1	6.8	0	0
	0.04	0	10	3.3	7.5	0	0
	0.05	0	9.2	3.7	7.3	0	0
	0.06	0	10.2	5.2	9.3	0	0

### Power and type I error (real genotypes)

**Figure 13:**
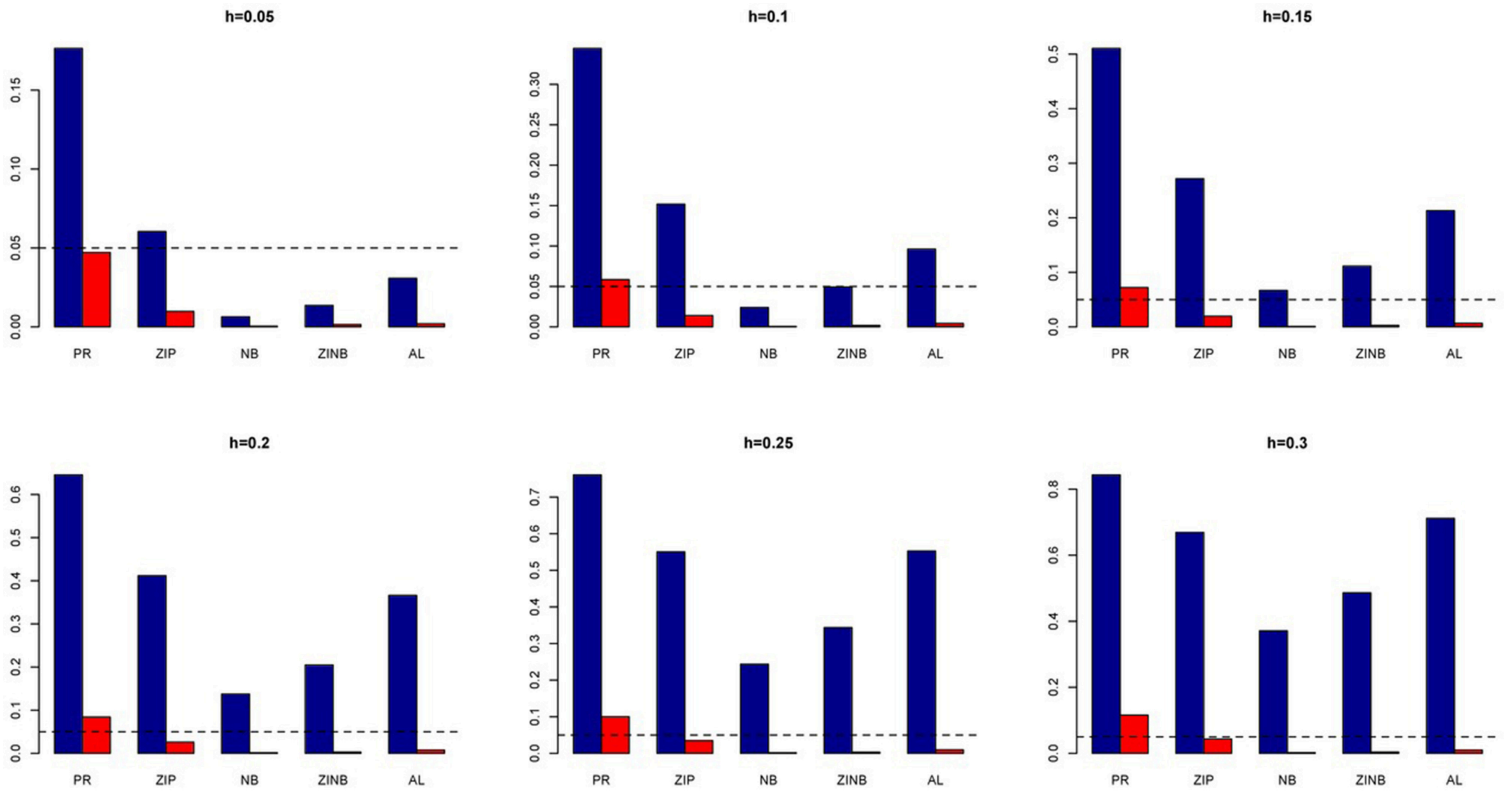
**Average Type I Error Rates (Red Bar) and Average Power (Blue Bar) from 1000 Replications for Colorectal Cancer Simulation Study**. Five methods are displayed from left to right: Poisson Regression (PR), Zero-inflated Poisson (ZIP) regression, Negative Binomial (NB) regression, Zero-inflated Negative Binomial (ZINB) regression, and adaptive LASSO (AL) penalized NB regression. Six marginal variances (*h* = 0.05, 0.10, 0.15, 0.20, 0.25, 0.30) are presented.

**Figure 14:**
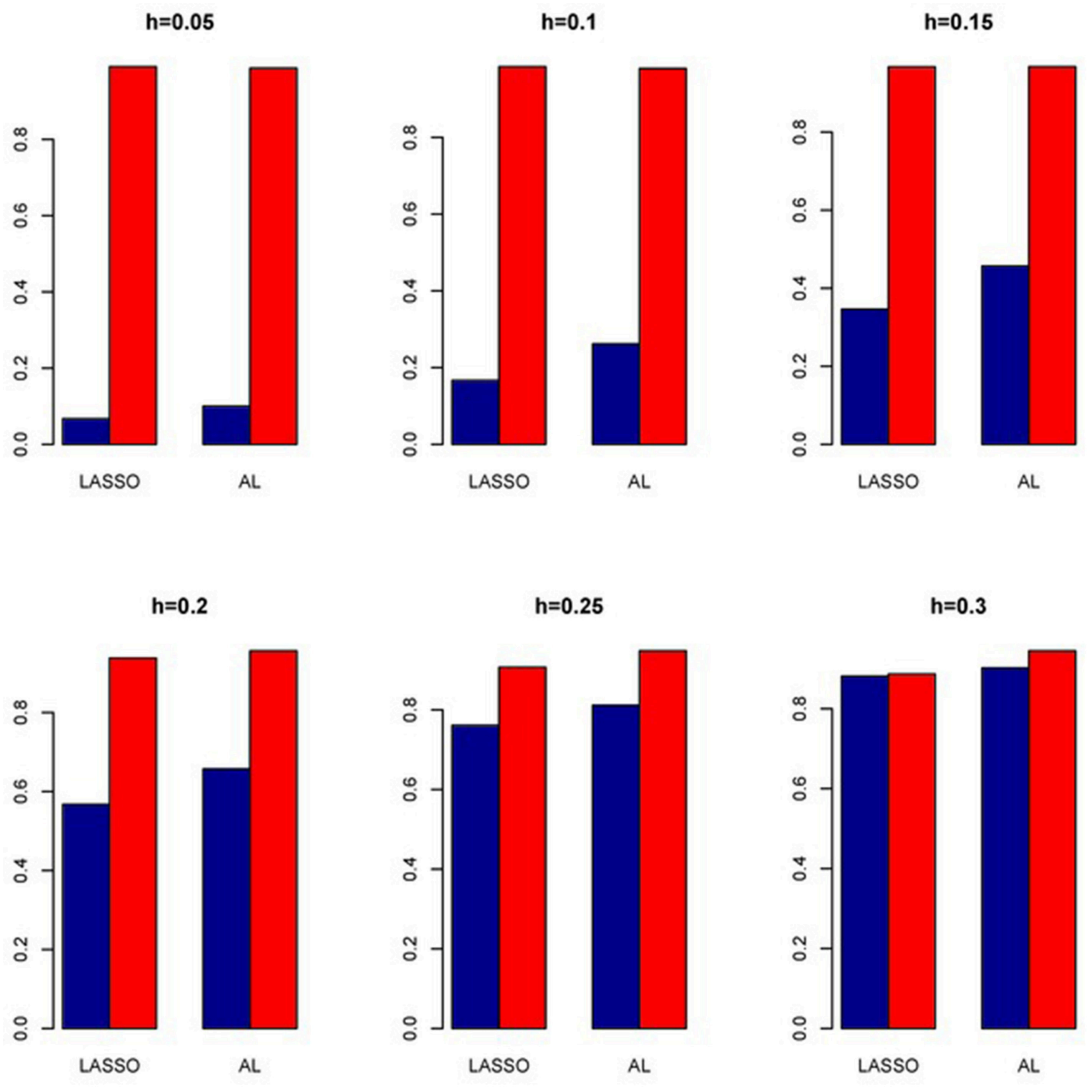
**Sensitivity (Red Bar) and Specificity (Blue Bar) from 1000 Replications for Colorectal Cancer Simulation Study**. Two methods are displayed: LASSO penalized NB regression (LASSO) and adaptive LASSO (AL) penalized NB regression. Six marginal variances (*h* = 0.05, 0.10, 0.15, 0.20, 0.25, 0.30) are presented.

We also investigate the empirical power of the five methods under consideration in additional simulations based on real genotype data as described above (Figure 13). It is interesting to note that the PR has the highest power in all the simulations with ZIP closely following next although both with slightly higher Type I error rates. In contrast, all the negative binomial-based methods have low Type I error rates. Among these NB-based methods, the proposed method has the highest empirical power across all situations. Here also we evaluated the penalized methods in terms of their sensitivity and specificity. It is evident from Figure 14 that the proposed AL method significantly outperforms the LASSO procedure with better sensitivity and similar specificity.

### Parameter estimation

We also compare the performance of the six methods with respect to parameter estimation. We measure the performance of each method based on the mean squared error (MSE) of its parameter estimates in the linear predictor scale. That is, for a method, suppose β^(*s*)^ is its estimate of the true β from dataset *s*, then its MSE is defined as

$$MSE=\sum_{s\;=\;1}^{1000}(\beta^{\left(s\right)}-\beta {)}^{\prime }(\beta^{\left(s\right)}-\beta ).$$

As shown in Tables 3–6, for all values of ρ, the penalized methods significantly outperform the non-penalized methods in terms of prediction accuracy. This confirms the advantage of the penalized regression, for parameter estimation and thus outcome prediction in the presence of zero-inflated count phenotypes. Here also we calculate the MSEs by only considering the replicates that converged. For a complete evaluation, we also calculate the MSEs for these three methods by considering both convergent and non-convergent replicates, and reach the same conclusion. Both LASSO methods have similar prediction accuracy. Hence, the analyses show strong support for the use of the proposed method.

**Table 3:** **The Mean Squared Errors (MSEs) of the Parameter Estimates Based on 1000 Replicates for Independent SNPs**.

**Sample Size**	**Marginal Variance**	**PR**	**ZIP**	**NB**	**ZINB**	**LASSO**	**AL**
*n* = 200	0.01	2.84	749.59	12.51	346.94	0.01	0.01
	0.02	2.84	842.08	12.51	332.52	0.02	0.03
	0.03	2.83	639.1	12.63	299.27	0.04	0.04
	0.04	2.82	737.22	13.28	364.73	0.05	0.05
	0.05	2.84	479.63	13.85	326.86	0.07	0.07
	0.06	2.9	520.9	15.09	352.02	0.08	0.09
*n* = 500	0.01	0.14	0.2	0.16	0.2	0.01	0.01
	0.02	0.14	0.2	0.16	0.2	0.02	0.02
	0.03	0.15	0.2	0.16	0.2	0.03	0.03
	0.04	0.15	0.2	0.17	0.2	0.04	0.04
	0.05	0.15	0.2	0.17	0.2	0.05	0.05
	0.06	0.17	0.2	0.19	0.2	0.06	0.05
*n* = 1000	0.01	0.06	0.07	0.07	0.07	0.01	0.01
	0.02	0.06	0.07	0.07	0.07	0.02	0.02
	0.03	0.06	0.07	0.07	0.07	0.03	0.02
	0.04	0.07	0.07	0.07	0.07	0.03	0.03
	0.05	0.07	0.07	0.07	0.07	0.04	0.03
	0.06	0.07	0.07	0.07	0.07	0.04	0.02

**Table 4:** **The Mean Squared Errors (MSEs) of the Parameter Estimates Based on 1000 Replicates for SNPs in Moderate LD**.

**Sample Size**	**Marginal Variance**	**PR**	**ZIP**	**NB**	**ZINB**	**LASSO**	**AL**
*n* = 200	0.01	2.92	542.39	12.18	326.83	0.01	0.01
	0.02	2.93	291.32	12.56	288.1	0.02	0.03
	0.03	2.9	330.95	12.49	186.88	0.04	0.04
	0.04	2.93	539.92	13.67	252.26	0.05	0.05
	0.05	2.97	337.68	14.2	813.24	0.07	0.07
	0.06	2.98	424.8	14.47	265.29	0.08	0.09
*n* = 500	0.01	0.16	0.23	0.18	0.23	0.01	0.01
	0.02	0.16	0.23	0.18	0.23	0.02	0.02
	0.03	0.16	0.23	0.18	0.23	0.03	0.03
	0.04	0.17	0.23	0.21	0.23	0.05	0.04
	0.05	0.18	0.23	0.21	0.23	0.06	0.05
	0.06	0.19	0.24	0.28	0.23	0.06	0.06
*n* = 1000	0.01	0.07	0.07	0.07	0.07	0.01	0.01
	0.02	0.07	0.07	0.07	0.07	0.02	0.02
	0.03	0.07	0.07	0.07	0.07	0.03	0.03
	0.04	0.07	0.07	0.07	0.07	0.03	0.03
	0.05	0.08	0.08	0.07	0.07	0.04	0.03
	0.06	0.08	0.08	0.08	0.07	0.04	0.02

**Table 5:** **The Mean Squared Errors (MSEs) of the Parameter Estimates Based on 1000 Replicates for SNPs in High LD**.

**Sample Size**	**Marginal Variance**	**PR**	**ZIP**	**NB**	**ZINB**	**LASSO**	**AL**
*n* = 200	0.01	25319.77	44548.96	113.34	31530.87	0.01	0.01
	0.02	18119.28	36267.35	206.43	24101.56	0.03	0.03
	0.03	26.05	16570.53	43.01	4579.47	0.04	0.04
	0.04	38.17	16627.73	44.04	4696.22	0.05	0.06
	0.05	38.46	15896.03	46.58	4592.57	0.07	0.07
	0.06	436.29	2521216	727.7	5014.9	0.09	0.09
*n* = 500	0.01	0.31	0.72	0.39	0.7	0.01	0.01
	0.02	0.32	0.7	0.4	0.67	0.02	0.02
	0.03	0.32	0.69	0.41	0.67	0.04	0.04
	0.04	0.33	0.68	0.41	0.67	0.05	0.05
	0.05	0.35	0.71	0.43	0.74	0.06	0.06
	0.06	0.36	0.68	0.44	0.67	0.07	0.07
*n* = 1000	0.01	0.13	0.16	0.15	0.16	0.01	0.01
	0.02	0.13	0.16	0.15	0.16	0.02	0.02
	0.03	0.13	0.16	0.15	0.16	0.03	0.03
	0.04	0.14	0.16	0.16	0.16	0.04	0.03
	0.05	0.15	0.16	0.16	0.15	0.04	0.04
	0.06	0.16	0.16	0.16	0.15	0.05	0.03

**Table 6:** **The Mean Squared Errors (MSEs) of the Parameter Estimates Based on 1000 Replicates for Colorectal Cancer Data**.

**Marginal Variance**	**PR**	**ZIP**	**NB**	**ZINB**	**LASSO**	**AL**
0.01	0.03	0.03	0.04	0.03	0.01	0.01
0.02	0.04	0.03	0.04	0.04	0.01	0.01
0.03	0.04	0.04	0.04	0.04	0.02	0.01
0.04	0.04	0.04	0.04	0.04	0.02	0.01
0.05	0.04	0.04	0.04	0.04	0.02	0.01
0.06	0.11	0.12	0.11	0.11	0.02	0.01

## Discussion

In this paper, we have proposed a novel variable selection method for detecting SNPs associated with zero-inflated count phenotypes in a negative binomial regression framework. We have considered a computationally efficient EM algorithm, which can simultaneously include a large number of genetic and environmental variables in the model. We have shown the superior performance of the proposed method through extensive simulation studies. Despite their wide applications in other scientific disciplines (Buu et al., 2011; Tang et al., 2014; Wang et al., 2014, 2015), zero-inflated count models are less thoroughly studied in genetics. We attempt to bridge this gap by investigating several state-of-the-art methods in light of their performance with respect to model misspecification and variable selection in association studies. As an example, two most popular approaches for handling zero-inflated count phenotypes in genetic association studies include the PR and the ZIP regression. However, it is unclear whether these methods provide sufficient power to detect a causal SNP when a model is misspecified. By simulating data under a variety of disease models, we have shown that using these methods in the presence of severe over dispersion can be misleading, as they tend to provide unprecedentedly high type I errors. In such situations, alternative methods based on the negative binomial regression framework (viz. the NB regression, the ZINB regression, and the adaptive LASSO penalized ZINB regression) provide better flexibility in modeling the over dispersion as compared to their Poisson counterparts. A similar conclusion was found in a recent article by Xu et al. (2015) in the context of microbiome data. Our results provide further insight on the empirical power of these alternative approaches for a range of variance values, which can guide researchers in designing studies involving zero-inflated count phenotypes. It is to be noted that, the current results are only valid when the underlying true model is ZINB. In practical situations when little is known about the data generating process, the choice of a model should be based on the closest fit between the observed and the predicted values. Moreover, most practical data analysis in genetic association studies involve several pre-processing steps including variable transformations, coding of variables, removal of outliers, handling of missing data, etc. which should be properly applied to these methods to ensure accurate results and easy implementation.

A few limitations of our study should be noted. Although our method can handle a large number of genetic and environmental variables, we have not evaluated its performance in the presence of interactions. Analysis of high-order interactions is a challenging topic in high-dimensional genetic research (Yi, 2010) and therefore, evaluation of the proposed method and other existing approaches in the presence of gene-gene (GXG) and gene-environment (GXE) interactions needs further consideration. Also, we have not evaluated our method for the rare variants (MAF < 1%), which are likely to play an important role in the “missing heritability” that cannot be explained by common and uncommon variants (Zeggini, 2011). Recently, a number of penalized regression methods have been proposed to handle rare variants for linear and generalized linear models, most of which follow various collapsing strategies and implement various grouped penalized regression methods (Zhou et al., 2010; Ayers and Cordell, 2013). Computationally efficient extension of the proposed method to rare variants in the context of zero-inflated count outcomes needs further research.

Apart from the limitations described above, in our simulation studies, we have not estimated the dispersion parameter as it slows down the EM algorithm considerably. Since the proposed adaptive LASSO estimator is a two-step procedure (initially obtaining the weights and then refitting the model with the estimated weights), the estimation process for θ becomes more computationally intensive for the proposed method as compared to others. However, since in practice, we anticipate to estimate θ for better prediction accuracy, following the suggestion of one anonymous reviewer, we replicated our simulation studies to learn the impact of estimating the dispersion parameter on the overall conclusion. The detailed results are presented in the Supplementary File (Supplementary Figures S2–S15, Supplementary Tables S1–S4). No noticeable difference was observed.

With the recent technological advances, and the upcoming sequencing experiments, we have the potential to identify additional causal variants across the entire genome, which will give us a better understanding of the genetic basis of complex diseases, such as rheumatoid arthritis (RA; Viatte et al., 2013b). With such a big amount of data, it is quite possible that the number of variants is far exceeding than the number of subjects. Also, quite often it will be necessary to examine a large number of genetic variants and environmental factors in a joint model, for which, traditional methods such as the ZINB or ZIP can be overwhelmed. For such situations, the proposed method provides a good alternative for conducting multi-SNP modeling in the presence of severe data collinearity induced by multiple highly linked variants.

## Author contributions

HM conceived the idea, performed the analysis, and wrote the manuscript; HT conceived the idea, designed the model, and wrote the manuscript. All authors read and approved the final manuscript.

### Conflict of interest statement

The authors declare that the research was conducted in the absence of any commercial or financial relationships that could be construed as a potential conflict of interest.
