# Supplementary material for: EM Adaptive LASSO—A Multilocus Modeling Strategy for Detecting SNPs Associated with Zero-inflated Count Phenotypes
Source: Front Genet. 2016 Mar 30;7:32. doi: 10.3389/fgene.2016.00032 (PMC4811966; doi:10.3389/fgene.2016.00032)
Supplement: Supplementary file 1 [file Presentation1.pdf]

Supplementary Materials for

"EM Adaptive LASSO - A Multilocus Modeling Framework for Associating SNPs with Zero-inflated Count Phenotypes" by

Himel Mallick[1,2], Hemant K. Tiwari[3]

[1]Harvard T. H. Chan School of Public Health, Boston, MA, USA

[2]Broad Institute of MIT and Harvard, Cambridge, MA, USA

[3]University of Alabama at Birmingham, Birmingham, AL, USA

Appendix A: Key Derivation of the Oracle Properties of the EM Adaptive LASSO Estimator for the ZINB Regression Model

Let $u_i = (X_i, y_i),\ i = 1, \ldots, n$ be independent and identically distributed observations from the ZINB distribution referenced in the main text. The log-likelihood function for the ZINB regression model is given by

$$L(\boldsymbol{\beta}, \boldsymbol{\gamma}; u_i) = \sum_{y_i=0} \log \left[\pi_i + (1-\pi_i) \left(\frac{\theta}{\theta + \mu_i} \right)^{\theta} \right] + \sum_{y_i>0} \log \left[(1-\pi_i) \left(\frac{\mu_i}{\theta + \mu_i} \right)^{y_i} \left(\frac{\theta}{\theta + \mu_i} \right)^{\theta} \right], \tag{1}$$

where $\mu_i = \exp(X_i \boldsymbol{\beta})$ and $\pi_i = \frac{\exp(X_i \boldsymbol{\gamma})}{1+\exp(X_i \boldsymbol{\gamma})}$, $i = 1, \ldots, n$.

Consider the penalized ZINB model with the adaptive LASSO penalty, which results from the following regularization problem

$$Q(\boldsymbol{\beta}, \boldsymbol{\gamma}) = -2 \log L(\boldsymbol{\beta}, \boldsymbol{\gamma}; u_i) + \lambda_{1n} \sum_{j=1}^{p} w_{1j} |\beta_j| + \lambda_{2n} \sum_{j=1}^{p} w_{2j} |\gamma_j|, \tag{2}$$

where $\boldsymbol{w_1} = (w_{11}, \ldots, w_{1p})'$ and $\boldsymbol{w_2} = (w_{21}, \ldots, w_{2p})'$ are known weight vectors, which are usually taken as the reciprocal of the unpenalized estimates obtained by maximizing (1), i.e. $\boldsymbol{w_1} = \frac{1}{\hat{\boldsymbol{\beta}}_{\text{MLE}}}$ and $\boldsymbol{w_2} = \frac{1}{\hat{\boldsymbol{\gamma}}_{\text{MLE}}}$.

We assume that the true model has a sparse representation. Let $\boldsymbol{\psi} = (\boldsymbol{\beta}^T, \boldsymbol{\gamma}^T)^T$. Let us denote the penalized maximum likelihood estimator as $\hat{\boldsymbol{\psi}} = (\hat{\boldsymbol{\beta}}^T, \hat{\boldsymbol{\gamma}}^T)^T$ and the true coefficient vector as $\boldsymbol{\psi_0} = (\boldsymbol{\beta_0}^T, \boldsymbol{\gamma_0}^T)^T$. Decompose $\boldsymbol{\psi_0} = (\boldsymbol{\psi_{10}}^T, \boldsymbol{\psi_{20}}^T)^T$ and assume that $\boldsymbol{\psi}_{20}^T$ contains all zero coefficients. Let us denote the subset of true nonzero coefficients as $\mathscr{A} = \{j : \psi_{j0} \neq 0\}$ and the subset of selected nonzero coefficients as $\hat{\mathscr{A}} = \{j : \hat{\psi}_j \neq 0\}$. We write the Fisher

information matrix

$$I(\boldsymbol{\psi_0}) = \begin{bmatrix} I_{11} & I_{12} \\ I_{21} & I_{22} \end{bmatrix},$$

where I_{11} is the Fisher information with the true nonzero submodel. The oracle property of ZINB with adaptive LASSO may be developed based on certain mild regularity conditions which are as follows:

(***A*1**): The Fisher information matrix $I(\boldsymbol{\psi})$ is finite and positive definite for all values of $\boldsymbol{\psi}$.

(***A*2**) There exists functions M_{jkl} such that

$$\left|\frac{\partial^3}{\partial\psi_j\partial\psi_k\partial\psi_l L(\boldsymbol{\psi}; u_i)}\right| \leq M_{jkl}(u_i) \text{ for all } \boldsymbol{\psi},$$

where $m_{jkl} = E_{\boldsymbol{\psi_0}}(M_{jkl}(u_i)) < \infty$ for all j, k, l.

Theorem 1: *Under assumptions (A1)–(A2), if* $\lambda_{1n} \to \infty$, $\lambda_{2n} \to \infty$, $\frac{\lambda_{1n}}{\sqrt{n}} \to 0$, $\frac{\lambda_{2n}}{\sqrt{n}} \to 0$, *then the estimator from the EM-adaptive LASSO method enjoys the oracle properties as follows:*

1. *Consistency in variable selection:* $\lim_n P(\hat{\mathscr{A}} = \mathscr{A}) = 1$, *and*

2. *Asymptotic normality of the nonzero coefficients:* $\sqrt{n}(\hat{\boldsymbol{\psi}} - \boldsymbol{\psi_0}) \to_d \mathscr{N}(\mathbf{0}, I_{11}^{-1})$

Proof: It is to be noted that the negative binomial distribution does not belong to the exponential family in a conventional sense, i.e. a negative binomial is a member of the GLM family only if θ is known and specified to the GLM family as a constant. Since, for a given value

of the dispersion parameter θ, the likelihood function in (1) can be decomposed into weighted logistic and negative binomial distributions (each belonging to the GLM family), Theorem 1 is the direct application of Theorem 4 in Zou [1]. Therefore, if $\lambda_{1n} \to \infty$, $\lambda_{2n} \to \infty$, $\frac{\lambda_{1n}}{\sqrt{n}} \to 0$, and $\frac{\lambda_{2n}}{\sqrt{n}} \to 0$, then the EM adaptive LASSO estimator for the ZINB model holds the oracle property: with probability tending to 1, the estimate of zero coefficients is 0, and the estimate for nonzero coefficients has an asymptotic normal distribution with mean being the true value and variance which approximately equals the submatrix of the Fisher information matrix containing nonzero coefficients.

References

[1] H. Zou. The adaptive LASSO and its oracle properties. *Journal of the American Statistical Association*, 101(476):1418–1429, 2006.

Appendix B: Additional Simulation Results

In this section, we present additional simulation studies in which **θ is estimated from the data**. The simulation setups are presented in the main text. Figure **S1** gives the heatmap of pairwise LD measurements for the 50 simulated SNPs in high LD scenario ($\rho = 0.9$). Figures **S2–S15** and Tables **S1–S4** describe the simulation results (in the same order as the main text).

Table S1: **The Mean Squared Errors (MSEs) of the Parameter Estimates Based on 1000 Replicates for Independent SNPs.**

Sample Size	Marginal Variance	PR	ZIP	NB	ZINB	LASSO	AL
$n = 200$	0.01	2.93	550.51	12.79	1391.33	0.01	0.01
	0.02	2.9	401.85	13.13	755.86	0.02	0.03
	0.03	2.92	453.45	14.09	468.22	0.04	0.04
	0.04	3.01	476.47	14.97	373.59	0.05	0.05
	0.05	3.05	695.69	15.45	913.49	0.07	0.07
	0.06	3.21	812.72	16.94	321.13	0.09	0.09
$n = 500$	0.01	0.15	0.21	0.17	0.21	0.01	0.01
	0.02	0.15	0.21	0.17	0.21	0.02	0.02
	0.03	0.15	0.21	0.17	0.2	0.03	0.03
	0.04	0.16	0.2	0.17	0.2	0.05	0.04
	0.05	0.16	0.2	0.17	0.2	0.06	0.05
	0.06	0.16	0.2	0.17	0.2	0.07	0.06
$n = 1000$	0.01	0.06	0.07	0.07	0.07	0.01	0.01
	0.02	0.06	0.07	0.07	0.07	0.02	0.02
	0.03	0.06	0.07	0.07	0.07	0.03	0.03
	0.04	0.07	0.07	0.07	0.07	0.04	0.03
	0.05	0.07	0.07	0.07	0.07	0.04	0.03
	0.06	0.07	0.07	0.07	0.07	0.04	0.02

Table S2: **The Mean Squared Errors (MSEs) of the Parameter Estimates Based on 1000 Replicates for SNPs in Moderate LD.**

Sample Size	Marginal Variance	PR	ZIP	NB	ZINB	LASSO	AL
$n = 200$	0.01	2.92	345.33	12.14	190.99	0.01	0.01
	0.02	2.88	448.41	12.05	390.46	0.02	0.03
	0.03	2.84	746.95	12.44	295.17	0.04	0.04
	0.04	2.89	581.88	13.4	304.52	0.05	0.06
	0.05	2.7	258.93	12.7	220.43	0.07	0.07
	0.06	2.79	405.49	13.64	266.28	0.09	0.09
$n = 500$	0.01	0.16	0.24	0.25	0.24	0.01	0.01
	0.02	0.16	0.23	0.21	0.23	0.02	0.02
	0.03	0.17	0.23	0.25	0.23	0.04	0.03
	0.04	0.17	0.23	0.21	0.23	0.05	0.04
	0.05	0.19	0.24	0.3	0.24	0.06	0.05
	0.06	0.19	0.24	0.31	0.24	0.07	0.06
$n = 1000$	0.01	0.07	0.07	0.07	0.07	0.01	0.01
	0.02	0.07	0.07	0.07	0.07	0.02	0.02
	0.03	0.07	0.07	0.07	0.07	0.03	0.03
	0.04	0.07	0.07	0.07	0.07	0.04	0.03
	0.05	0.08	0.07	0.07	0.07	0.04	0.03
	0.06	0.08	0.08	0.08	0.07	0.04	0.02

Table S3: **The Mean Squared Errors (MSEs) of the Parameter Estimates Based on 1000 Replicates for SNPs in High LD.**

Sample Size	Marginal Variance	PR	ZIP	NB	ZINB	LASSO	AL
	0.01	428.54	16579.94	1117.71	3564.08	0.01	0.01
	0.02	52.07	14735.95	30.99	3891.21	0.03	0.03
	0.03	26.99	16623.79	45.81	5619.53	0.04	0.04
$n = 200$	0.04	24.72	15070.89	42.61	5505.69	0.06	0.06
	0.05	25.35	19526.84	45.49	3876.16	0.07	0.08
	0.06	7.93	14418.32	24.54	5062.99	0.09	0.09
	0.01	0.31	0.74	0.4	0.73	0.01	0.01
	0.02	0.31	0.69	0.4	0.71	0.02	0.02
	0.03	0.32	0.7	0.41	0.69	0.04	0.04
$n = 500$	0.04	0.33	0.7	0.42	0.71	0.05	0.05
	0.05	0.35	0.74	0.45	0.74	0.06	0.06
	0.06	0.36	0.71	0.43	0.72	0.08	0.07
	0.01	0.13	0.16	0.15	0.16	0.01	0.01
	0.02	0.13	0.16	0.15	0.16	0.02	0.02
	0.03	0.13	0.16	0.16	0.16	0.03	0.03
$n = 1000$	0.04	0.14	0.16	0.16	0.16	0.04	0.03
	0.05	0.15	0.16	0.16	0.15	0.05	0.04
	0.06	0.16	0.16	0.16	0.15	0.05	0.04

Table S4: **The Mean Squared Errors (MSEs) of the Parameter Estimates Based on 1000 Replicates for Colorectal Cancer Simulation Study.**

Marginal Variance	PR	ZIP	NB	ZINB	LASSO	AL
0.01	0.03	0.03	0.04	0.03	0.01	0.01
0.02	0.04	0.03	0.04	0.04	0.01	0.01
0.03	0.04	0.04	0.04	0.04	0.02	0.01
0.04	0.04	0.04	0.04	0.04	0.02	0.02
0.05	0.04	0.04	0.04	0.04	0.02	0.01
0.06	0.11	0.12	0.11	0.11	0.02	0.01

B

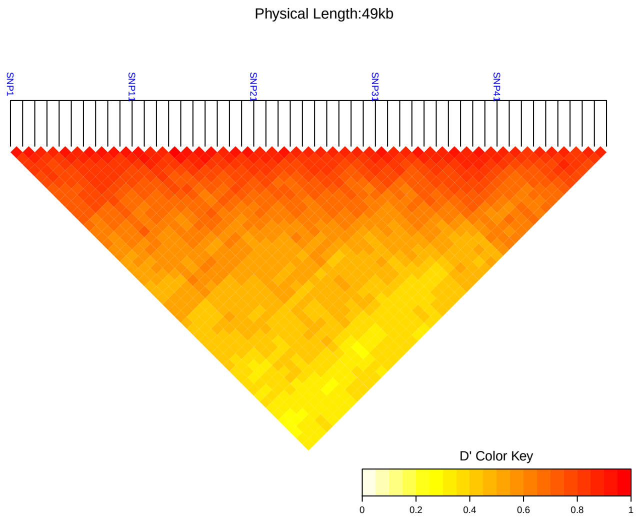

Figure S1: **Heatmap of Pairwise LD Measurements for the 50 Simulated SNPs in High LD Scenario.**

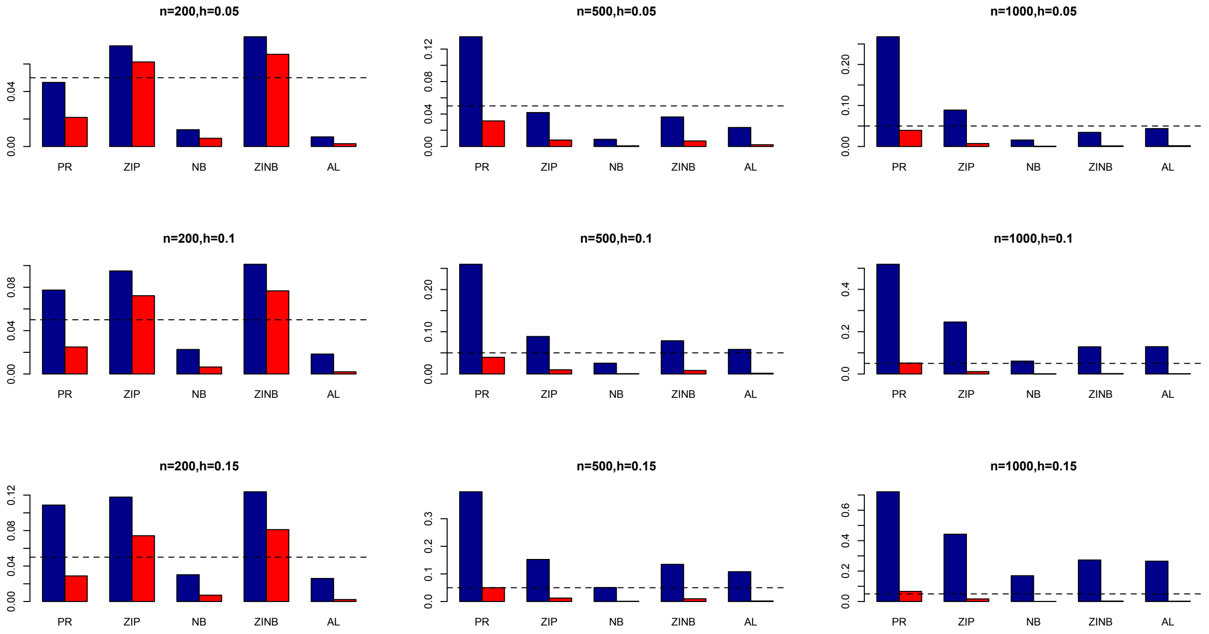

Figure S2: **Average Type I Error Rates (Red Bar) and Average Power (Blue Bar) from 1000 Replications for Independents SNPs ($\boldsymbol{\rho = 0}$)**. Five methods are displayed from left to right: Poisson Regression (PR), Zero-inflated Poisson (ZIP) regression, Negative Binomial (NB) regression, Zero-inflated Negative Binomial (ZINB) regression, and adaptive LASSO (AL) penalized NB regression. Three sample sizes ($n = 200, 500, 1000$) (in rows) and three marginal variances ($h = 0.05, 0.10, 0.15$) (in columns) are presented.

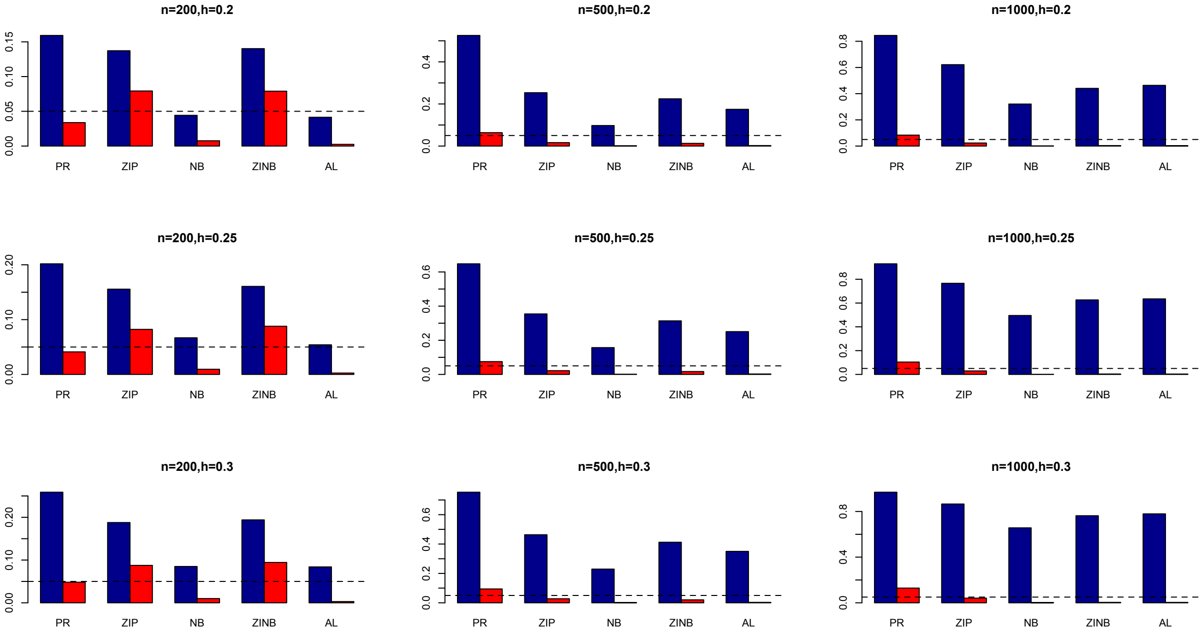

Figure S3: **Average Type I Error Rates (Red Bar) and Average Power (Blue Bar) from 1000 Replications for Independents SNPs ($\boldsymbol{\rho = 0}$)**. Five methods are displayed from left to right: Poisson Regression (PR), Zero-inflated Poisson (ZIP) regression, Negative Binomial (NB) regression, Zero-inflated Negative Binomial (ZINB) regression, and adaptive LASSO (AL) penalized NB regression. Three sample sizes ($n = 200, 500, 1000$) (in rows) and three marginal variances ($h = 0.20, 0.25, 0.30$) (in columns) are presented.

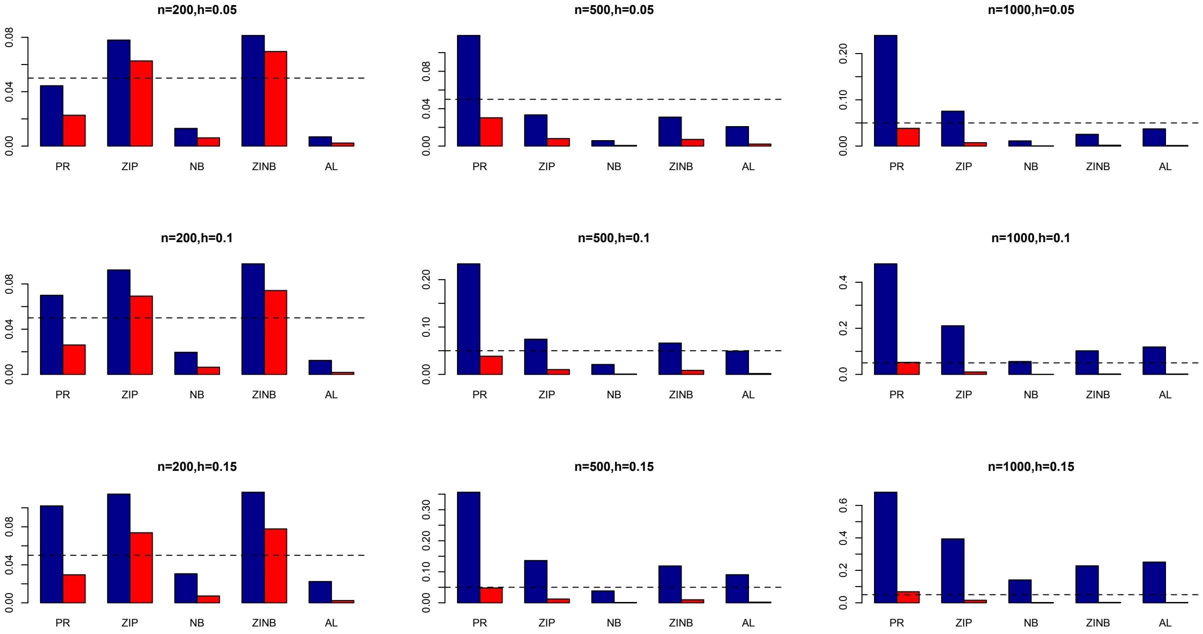

Figure S4: **Average Type I Error Rates (Red Bar) and Average Power (Blue Bar) from 1000 Replications for SNPs in Moderate LD ($\rho = 0.5$)**. Five methods are displayed from left to right: Poisson Regression (PR), Zero-inflated Poisson (ZIP) regression, Negative Binomial (NB) regression, Zero-inflated Negative Binomial (ZINB) regression, and adaptive LASSO (AL) penalized NB regression. Three sample sizes ($n = 200, 500, 1000$) (in rows) and three marginal variances ($h = 0.05, 0.10, 0.15$) (in columns) are presented.

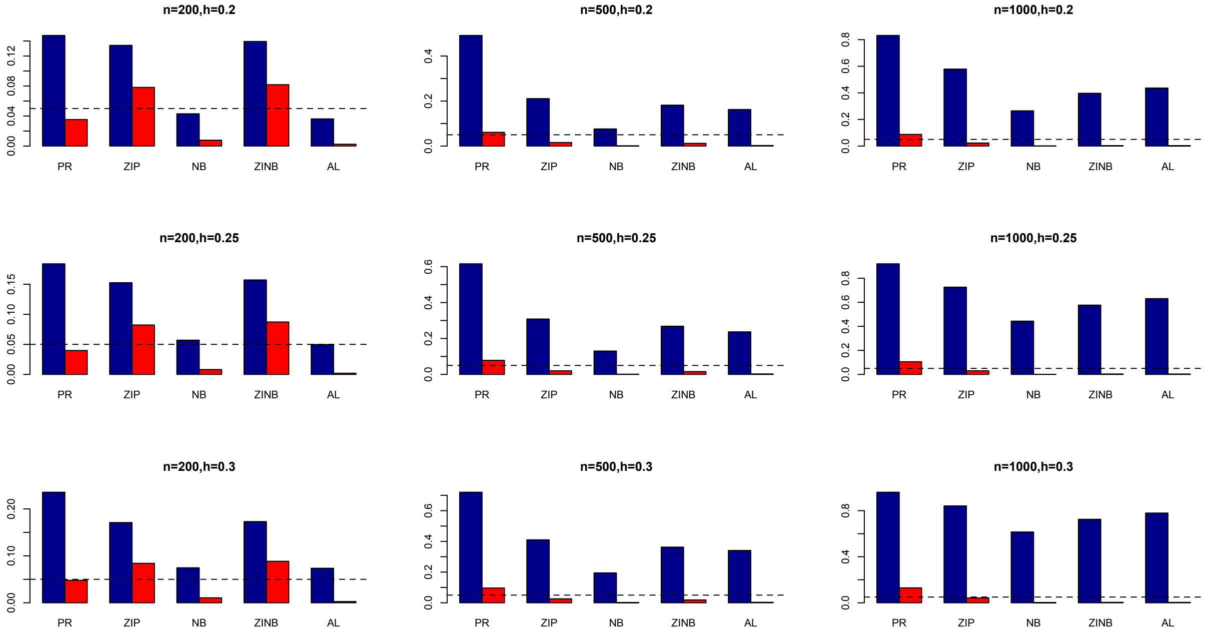

Figure S5: **Average Type I Error Rates (Red Bar) and Average Power (Blue Bar) from 1000 Replications for SNPs in Moderate LD ($\rho = 0.5$)**. Five methods are displayed from left to right: Poisson Regression (PR), Zero-inflated Poisson (ZIP) regression, Negative Binomial (NB) regression, Zero-inflated Negative Binomial (ZINB) regression, and adaptive LASSO (AL) penalized NB regression. Three sample sizes ($n = 200, 500, 1000$) (in rows) and three marginal variances ($h = 0.20, 0.25, 0.30$) (in columns) are presented.

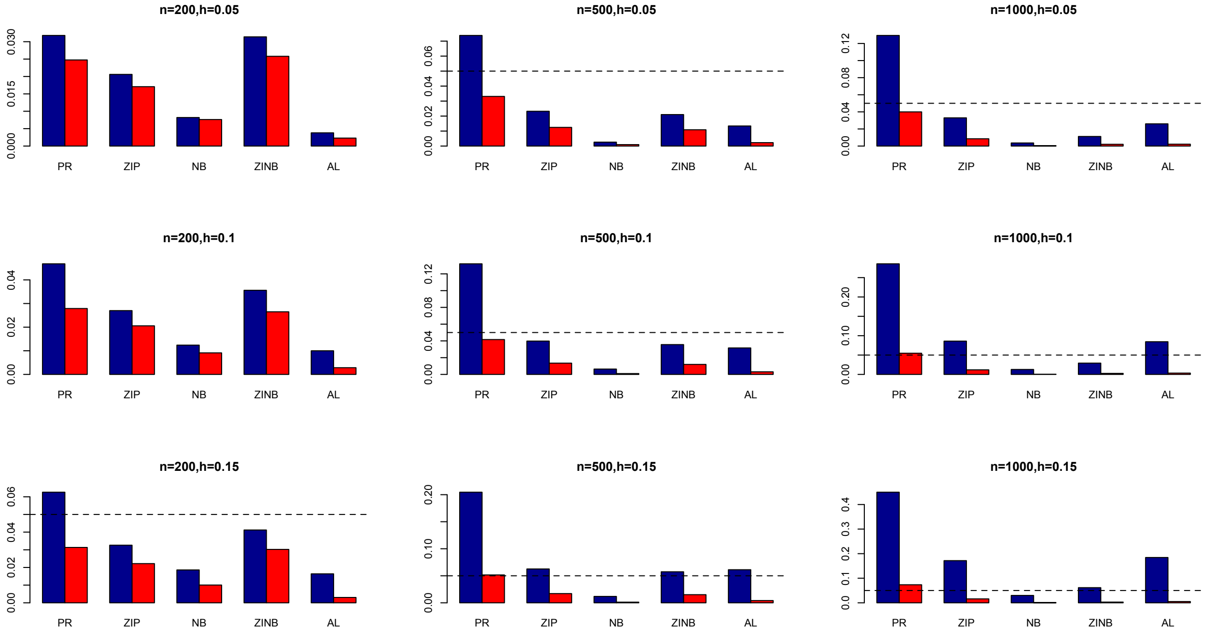

Figure S6: **Average Type I Error Rates (Red Bar) and Average Power (Blue Bar) from 1000 Replications for SNPs in High LD ($\rho = 0.9$)**. Five methods are displayed from left to right: Poisson Regression (PR), Zero-inflated Poisson (ZIP) regression, Negative Binomial (NB) regression, Zero-inflated Negative Binomial (ZINB) regression, and adaptive LASSO (AL) penalized NB regression. Three sample sizes ($n = 200, 500, 1000$) (in rows) and three marginal variances ($h = 0.05, 0.10, 0.15$) (in columns) are presented.

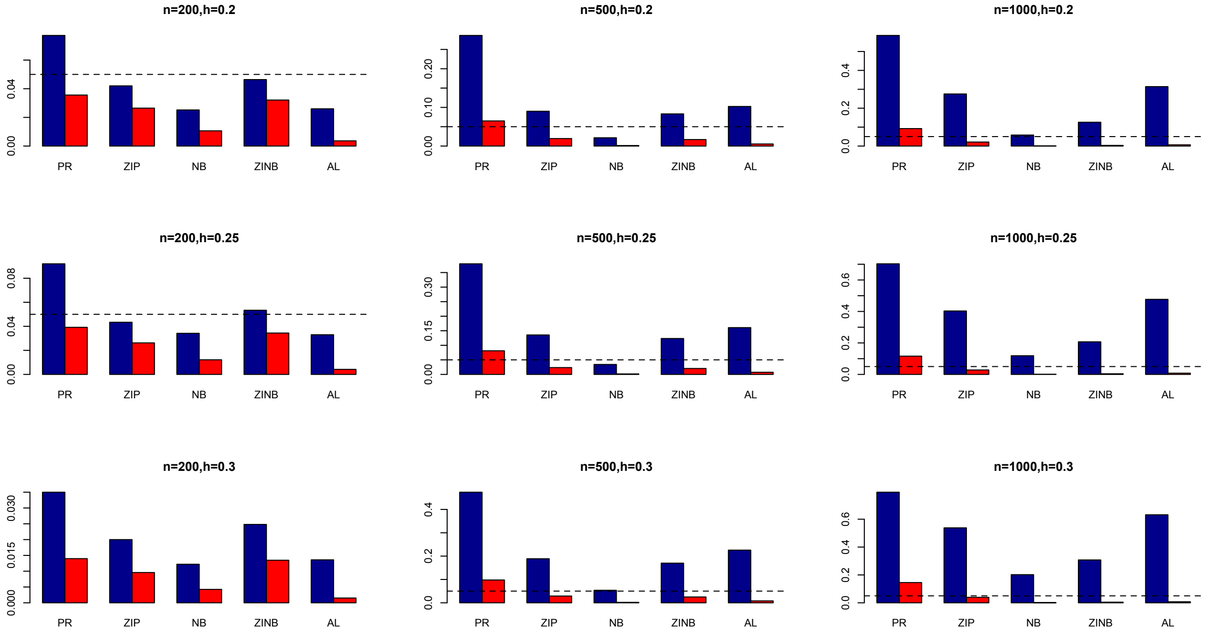

Figure S7: **Average Type I Error Rates (Red Bar) and Average Power (Blue Bar) from 1000 Replications for SNPs in High LD ($\rho = 0.9$)**. Five methods are displayed from left to right: Poisson Regression (PR), Zero-inflated Poisson (ZIP) regression, Negative Binomial (NB) regression, Zero-inflated Negative Binomial (ZINB) regression, and adaptive LASSO (AL) penalized NB regression. Three sample sizes ($n = 200, 500, 1000$) (in rows) and three marginal variances ($h = 0.20, 0.25, 0.30$) (in columns) are presented.

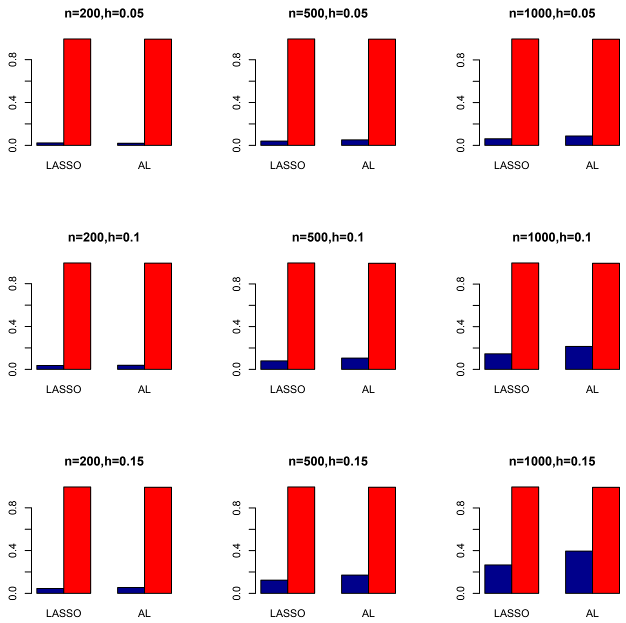

Figure S8: **Sensitivity (Red Bar) and Specificity (Blue Bar) from 1000 Replications for Independents SNPs ($\rho = 0$).** Two methods are displayed: LASSO penalized NB regression (LASSO) and adaptive LASSO (AL) penalized NB regression. Three sample sizes ($n = 200, 500, 1000$) (in rows) and three marginal variances ($h = 0.05, 0.10, 0.15$) (in columns) are presented.

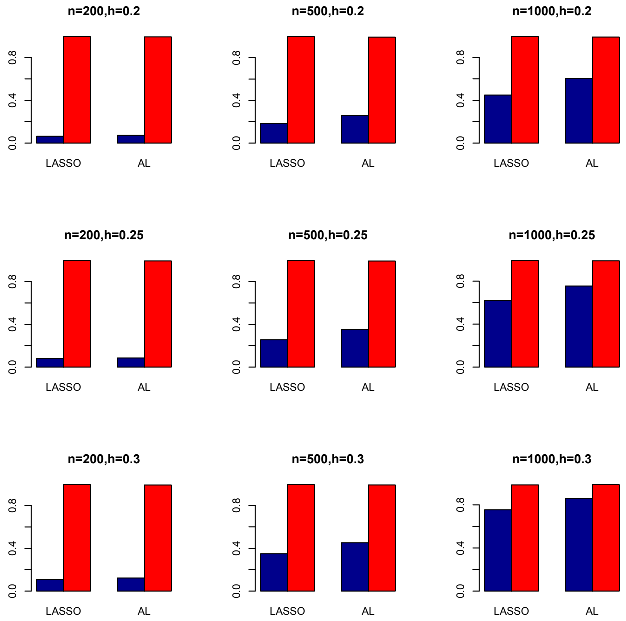

Figure S9: **Sensitivity (Red Bar) and Specificity (Blue Bar) from 1000 Replications for Independents SNPs ($\rho = 0$).** Two methods are displayed: LASSO penalized NB regression (LASSO) and adaptive LASSO (AL) penalized NB regression. Three sample sizes ($n = 200, 500, 1000$) (in rows) and three marginal variances ($h = 0.20, 0.25, 0.30$) (in columns) are presented.

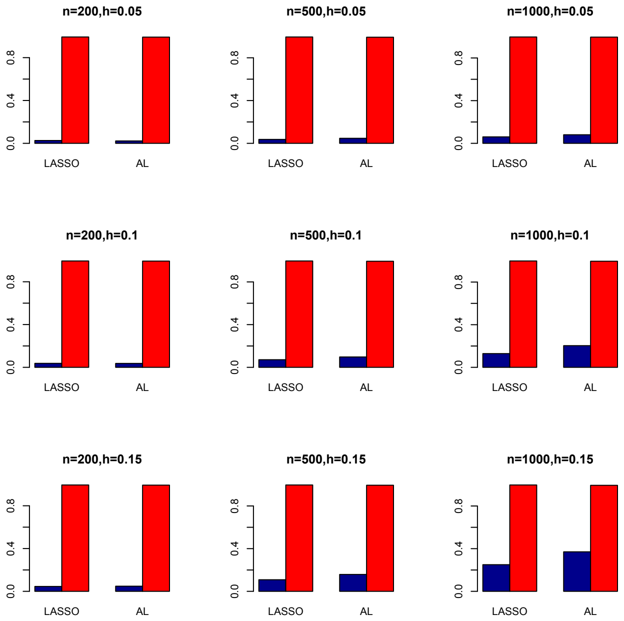

Figure S10: **Sensitivity (Red Bar) and Specificity (Blue Bar) from 1000 Replications for SNPs in Moderate LD ($\rho = 0.5$).** Two methods are displayed: LASSO penalized NB regression (LASSO) and adaptive LASSO (AL) penalized NB regression. Three sample sizes ($n = 200, 500, 1000$) (in rows) and three marginal variances ($h = 0.05, 0.10, 0.15$) (in columns) are presented.

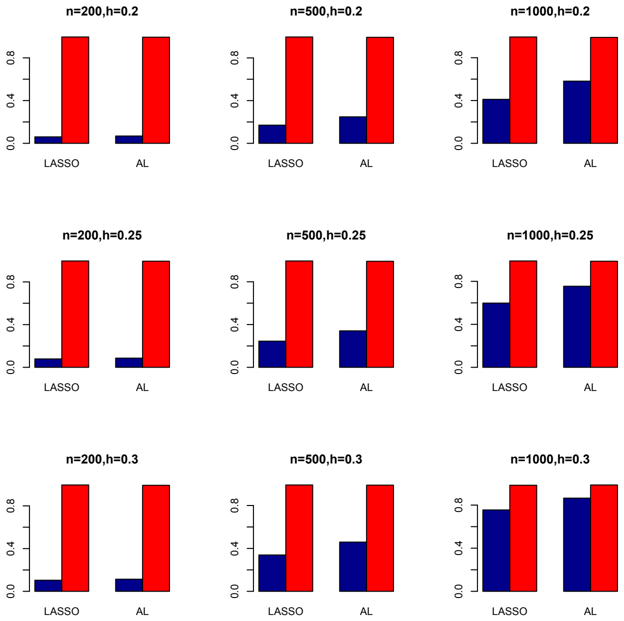

Figure S11: **Sensitivity (Red Bar) and Specificity (Blue Bar) from 1000 Replications for SNPs in Moderate LD ($\rho = 0.5$).** Two methods are displayed: LASSO penalized NB regression (LASSO) and adaptive LASSO (AL) penalized NB regression. Three sample sizes ($n = 200, 500, 1000$) (in rows) and three marginal variances ($h = 0.20, 0.25, 0.30$) (in columns) are presented.

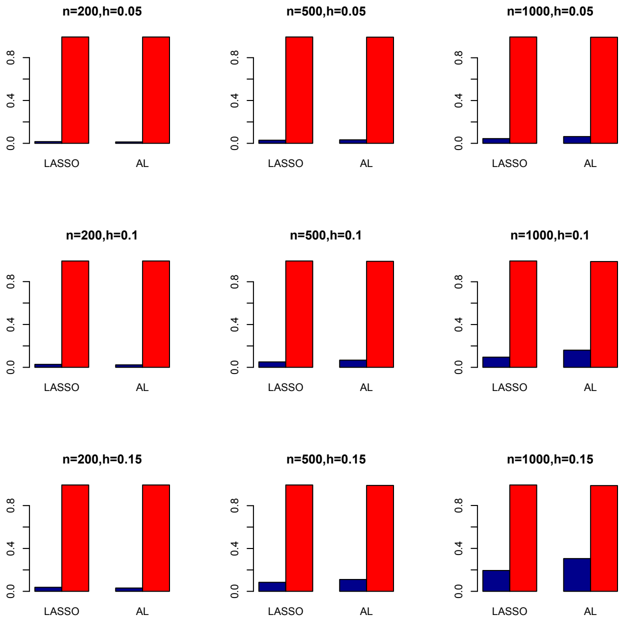

Figure S12: **Sensitivity (Red Bar) and Specificity (Blue Bar) from 1000 Replications for SNPs in High LD ($\rho = 0.9$)**. Two methods are displayed: LASSO penalized NB regression (LASSO) and adaptive LASSO (AL) penalized NB regression. Three sample sizes ($n = 200, 500, 1000$) (in rows) and three marginal variances ($h = 0.05, 0.10, 0.15$) (in columns) are presented.

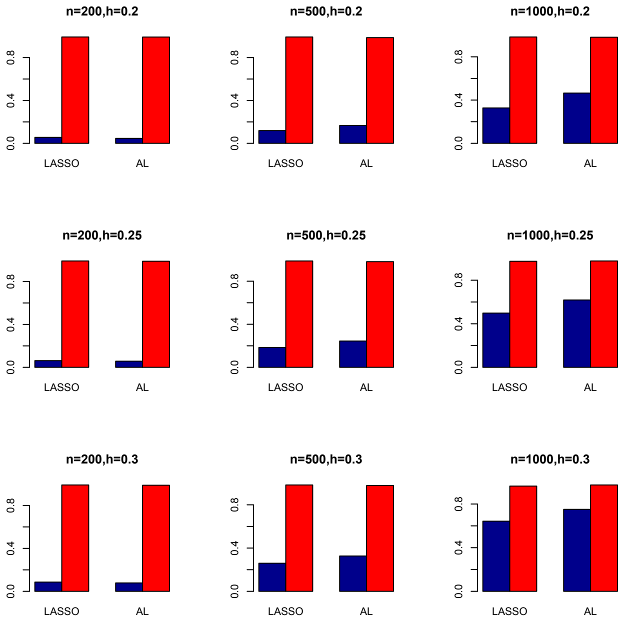

Figure S13: **Sensitivity (Red Bar) and Specificity (Blue Bar) from 1000 Replications for SNPs in High LD ($\rho = 0.9$)**. Two methods are displayed: LASSO penalized NB regression (LASSO) and adaptive LASSO (AL) penalized NB regression. Three sample sizes ($n = 200, 500, 1000$) (in rows) and three marginal variances ($h = 0.20, 0.25, 0.30$) (in columns) are presented.

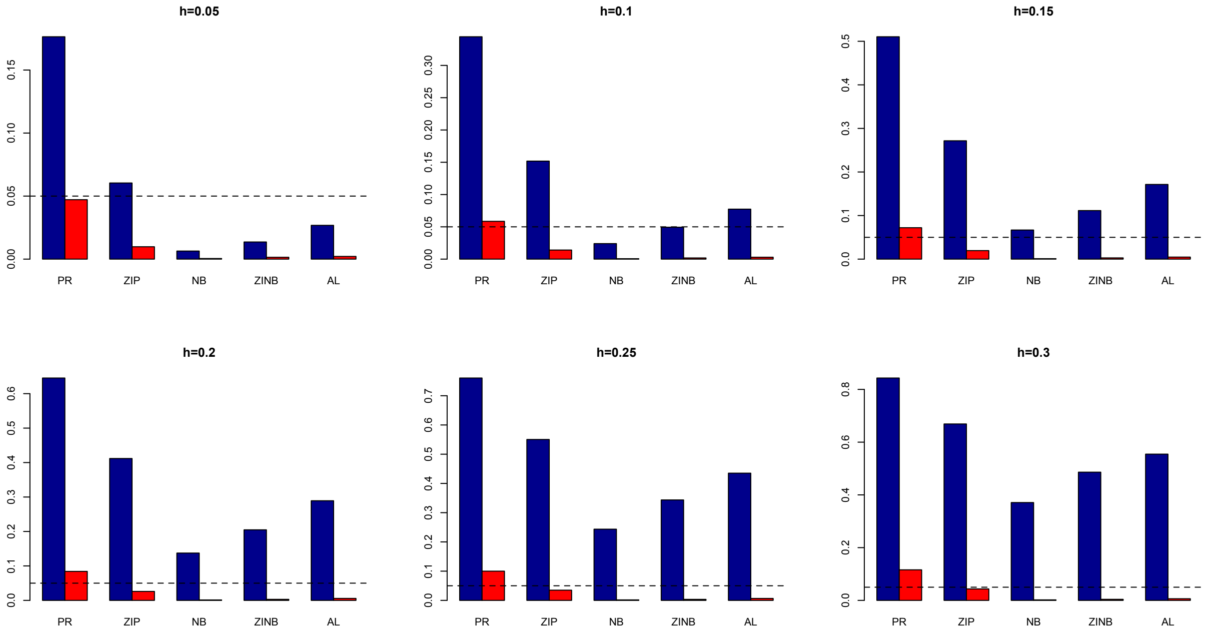

Figure S14: **Average Type I Error Rates (Red Bar) and Average Power (Blue Bar) from 1000 Replications for Colorectal Cancer Simulation Study**. Five methods are displayed from left to right: Poisson Regression (PR), Zero-inflated Poisson (ZIP) regression, Negative Binomial (NB) regression, Zero-inflated Negative Binomial (ZINB) regression, and adaptive LASSO (AL) penalized NB regression. Six marginal variances ($h = 0.05, 0.10, 0.15, 0.20, 0.25, 0.30$) are presented.

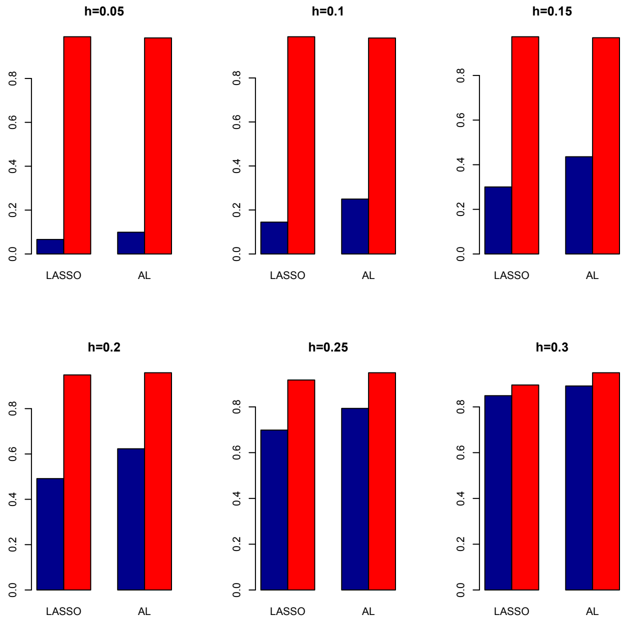

Figure S15: **Sensitivity (Red Bar) and Specificity (Blue Bar) from 1000 Replications for Colorectal Cancer Simulation Study.** Two methods are displayed: LASSO penalized NB regression (LASSO) and adaptive LASSO (AL) penalized NB regression. Six marginal variances ($h = 0.05, 0.10, 0.15, 0.20, 0.25, 0.30$) are presented.
